# Synthesis and QSAR Study of Novel 6-Chloro-3-(2-Arylmethylene-1-methylhydrazino)-1,4,2-benzodithiazine 1,1-Dioxide Derivatives with Anticancer Activity

**DOI:** 10.3390/molecules20045754

**Published:** 2015-04-01

**Authors:** Jarosław Sławiński, Beata Żołnowska, Zdzisław Brzozowski, Anna Kawiak, Mariusz Belka, Tomasz Bączek

**Affiliations:** 1 Department of Organic Chemistry, Medical University of Gdańsk, Al. Gen. J. Hallera 107, 80-416 Gdańsk, Poland; E-Mails: zolnowska@gumed.edu.pl (B.Ż.); brzozowskigumed@gmail.com (Z.B.); 2 Department of Biotechnology, Intercollegiate Faculty of Biotechnology, University of Gdańsk and Medical University of Gdańsk, ul. Kładki 24, 80-822 Gdańsk, Poland; E-Mail: kawiak@biotech.ug.pl; 3 Department of Human Physiology, Medical University of Gdańsk, ul. Tuwima 15, 80-210 Gdańsk, Poland; 4 Department of Pharmaceutical Chemistry, Medical University of Gdańsk, Al. Gen. J. Hallera 107, 80-416 Gdańsk, Poland; E-Mails: mariusz.belka@gumed.edu.pl (M.B.); tbaczek@gumed.edu.pl (T.B.)

**Keywords:** sulfonamides, 1,4,2-benzodithiazines, cytotoxic activity, anticancer activity, structure-activity relationship, QSAR, stepwise regression

## Abstract

A series of new 6-chloro-3-(2-arylmethylene-1-methylhydrazino)-1,4,2-benzodithiazine 1,1-dioxide derivatives were effectively synthesized from *N*-methyl-*N*-(6-chloro-1,1-dioxo-1,4,2-benzodithiazin-3-yl)hydrazines. The intermediate compounds as well as the products, were evaluated for their cytotoxic effects toward three human cancer cell lines. All compounds shown moderate or weak cytotoxic effects against the tested cancer cell lines, but selective cytotoxic effects were observed. Compound **16** exhibited the most potent cytotoxic activity against the HeLa cell line, with an IC_50_ value of 10 µM, while **14** was the most active against the MCF-7 and HCT-116 cell lines, affording IC_50_ values of 15 µM and 16 µM, respectively. The structure-activity relationship was evaluated based on QSAR methodology. The QSAR MCF-7 model indicated that natural charge on carbon atom C13 and energy of highest occupied molecular orbital (HOMO) are highly involved in cytotoxic activity against MCF-7 cell line. The cytotoxic activity of compounds against HCT-116 cell line is dependent on natural charge on carbon atom C13 and electrostatic charge on nitrogen atom N10. The obtained QSAR models could provide guidelines for further development of novel anticancer agents.

## 1. Introduction

**Figure 1:**
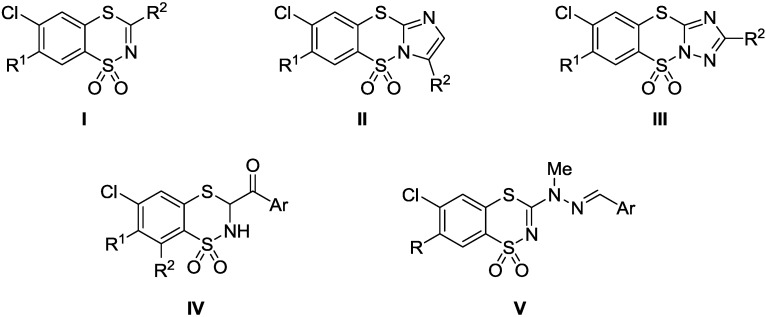
Chemical structures of biological active 1,1-dioxo-1,4,2-benzodithiazines **I**–**IV** [1,2,3,4,5,6,7,8,9,10,11,12,13,14,15], and **V**.

The 1,1-dioxo-1,4,2-benzodithiazines constitute a fundamental class of compounds in our laboratories since 1984, they have a wide range of biological activity and are useful substrates for many syntheses. It has been demonstrated that many 6-chloro-1,1-dioxo-1,4,2-benzodithiazine derivatives (**I**, Figure 1) possess low acute toxicity in mice and rats and depending on their structure, they act as potential diuretic [1,2,3,4,5,6] radioprotective [4], cholagogue [6,7], antiarrhythmic [4,6], or hypotensive [4,5,6,7] agents. It has also been shown that some 6-chloro-1,1-dioxo-1,4,2-benzodithiazines exhibit remarkable antitumor activities (**I** [8,9,10], **II** and **III** [10,11,12], Figure 1) or anti-HIV-1 activity (**I** and **IV** [13,14,15] Figure 1). This prompted us to investigate new potential antiproliferative agents with the general structure of type **V** (Figure 1) in which moieties attached to positions 3 and 7 of 6-chloro-1,1-dioxo-1,4,2-benzodithiazine ring were varied in molecular shape and electronic properties.

Thus, we synthesized new series of 6-chloro-7-R^1^-3-(2-arylmethylene-1-methylhydrazino)-1,1-dioxo-1,4,2-benzodithiazines **V** and evaluated their *in vitro* anticancer activity against human breast (MCF-7), colon (HCT-116) and cervical (HeLa) cancer cell lines. To explain how structural features influence the biological activities the quantitative structure-activity relationship (QSAR) method was applied [16].

## 2. Results and Discussion

### 2.1. Chemistry

**Scheme 1:**
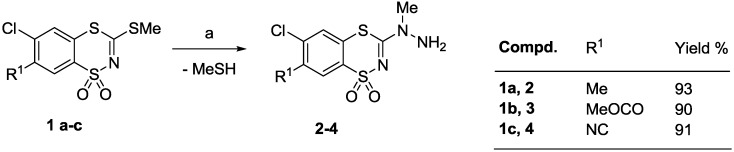
Synthesis of *N*-methyl-*N*-(6-chloro-1,1-dioxo-1,4,2-benzodithiazin-3-yl)hydrazines **2**‒**4**.

**Scheme 2:**
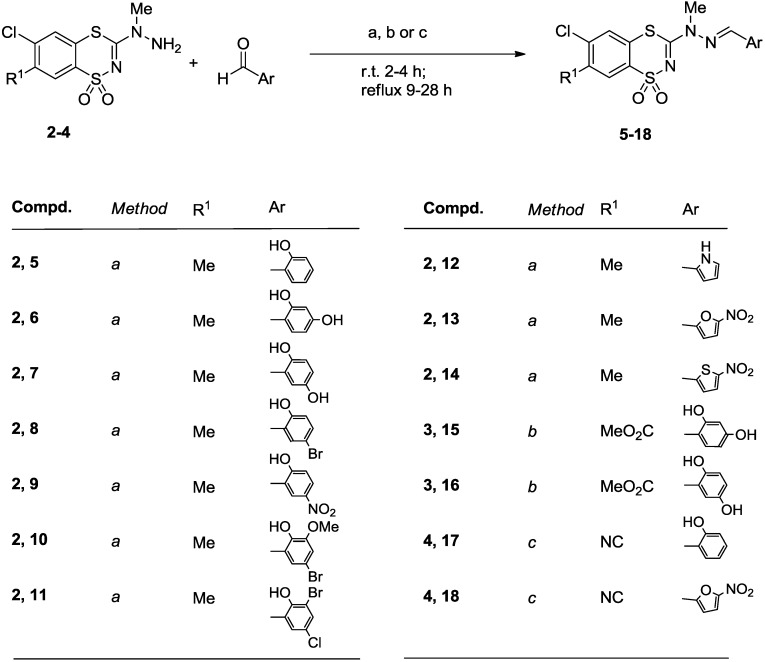
Synthesis of 6-chloro-7-R^1^-3-(2-arylmethylene-1-methylhydrazino)-1,1-dioxo-1,4,2-benzodithiazines **5**‒**18**.

A series of the desired 6-chloro-7-R^1^-3-(2-arylmethylene-1-methylhydrazino)-1,1-dioxo-1,4,2-benzodithiazine derivatives **5**‒**18** was prepared in a two-step process as shown in Scheme 1 and Scheme 2. Thus, the starting *N*-methyl-*N*-(6-chloro-7-R^1^-1,1-dioxo-1,4,2-benzodithiazin-3-yl)hydrazines **2**‒**4** were synthesized by the reaction of the appropriate 3-methylthiobenzodithiazines **1a**‒**c** with *N*-methylhydrazine in boiling dry acetonitrile (Scheme 1). It is well known that alkylations of alkyl-substituted hydrazines take place at the more substituted nitrogen atom [17,18,19], therefore in our investigations competition between *N*^1^ and *N*^2^ alkylation was not observed, and the regioselective substitution product, *i.e.*, *N*-methyl-*N*-substituted hydrazines, were obtanined in high yields (90%‒93%). Subsequent condensation of the hydrazine derivatives with an aldehyde in the presence of a catalytic amount of glacial acetic acid furnished the expected final products **5**‒**18** in good yields (87%‒98%) (Scheme 2).

*Reagents and conditions*: (**a**) methylhydrazine (1.04 M equiv.), dry acetonitrile, r.t. 8 h, reflux 10–16 h.

The structures of the compounds **2**‒**4** and the final substances **5**‒**18** were confirmed by IR, ^1^H-NMR and ^13^C-NMR spectroscopy. For example, in the ^1^H-NMR spectra, the presence of N-*CH*_3_ and N-*NH*_2_ groups in **2**–**4** was identified from the singlet signals at 3.36–3.35 and 5.72–5.85 ppm, respectively. Meanwhile, the appearance of N=CH signals at 8.21‒8.66 ppm in the spectra of **5**‒**18** proved the presence of this group and conformed the proposed structure of the final compounds.

### 2.2. Cytotoxic Activity

Compounds **6**‒**18** and the intermediates **2** and **4** were evaluated *in vitro* for their effects on the viability of three human cancer cell lines: MCF-7 (breast cancer), HCT-116 (colon cancer) and HeLa (cervical cancer). The concentration required for 50% inhibition of cell viability IC_50_ was calculated and compared with the reference drug cisplatin, and the results are given in Table 1.

The most active compounds possess at *N*^2^ of the 3-hydrazinobenzodithiazine scaffold a 2,4-dihydroxyphenyl (compound **6**) or 2-hydroxy-5-nitrophenyl (compound **9**) moiety and showed outstanding anticancer activity against all tested cell lines, whereas compounds **2**, **4**, **7**, **13** and **18** demonstrated relatively weak activity, with IC_50_ values over 100 µM.

*Reagents and conditions*: (**a**) appropriate aryl aldehyde (1.25 M equiv.), EtOH, AcOH (catalytic amount), r.t. 3 h, reflux 25 h; (**b**) appropriate aryl aldehyde (1.33 M equiv.), MeOH, AcOH (catalytic amount), r.t. 4 h, reflux 28 h; (**c**) appropriate aryl aldehyde (1.33 M equiv.), 2-methoxyethanol, AcOH (catalytic amount), r.t. 2 h, reflux 9 h.

Interestingly, compound **16** (*Ar* = 2,5-dihydroxyphenyl) exhibited the strongest cytotoxic activity against the HeLa cell line with an IC_50_ value of 10 µM, however shifting of the hydroxyl functionality from position 5 (compound **16**) to position 4 (compound **15**) on the phenyl ring caused the loss of cytotoxic activity (IC_50_ = 170 µM) against this cell line. On the other hand, the presence of *Ar* = 5-nitrothiophene ring (compound **14**) determined the highest cytotoxic activities against MCF-7 and HCT-116 cell lines, and affording the IC_50_ values of 15 µM and 16 µM, respectively, which changed significantly after its replacement by a 5-nitrofuran ring (compound **13**) resulting in poor activity (IC_50_ = 140 µM, MCF-7, and IC_50_ = 115 µM, HCT-116).

Nevertheless, considering the compounds’ activities against each single cell line, the nature of substituents R^1^ and *Ar* have varying influences on the biological activity of these compounds. For instance, cytotoxic activity in the series of *N*^2^-methylenesubstituted 3-hydrazino-7-methyl-1,1-dioxo-1,4,2-benzodithiazines against the MCF-7 cell line decreased in the following order: 5-nitrothiophenyl (**14**) > 2,4-dihydroxyphenyl (**6**) > pyrrolyl (**12**) > 3-bromo-5-chloro-2-hydroxyphenyl (**11**) ≈ 5-bromo-2-hydroxy-3-methoxyphenyl (**10**) > 5-bromo-2-hydroxyphenyl (**8**) > 2-hydroxy-5-nitrophenyl (**9**), suggesting the necessity to choose other and more objective methods of establishing the structure-activity relationships, such as QSAR methodology.

**Table 1:** Cytotoxicity of compounds **2**, **4** and **6**‒**18** toward human cancer cell lines.

Compounds	IC_50_ [µM]		
	MCF-7	HCT-116	HeLa
**2**	250 ± 7	280 ± 1	125 ± 7
**4**	155 ± 2	240 ± 5	99 ± 4
**6**	45 ± 0.5	31 ± 1	66 ± 2
**7**	270 ± 13	150 ± 4	>>100 ^a^
**8**	84 ± 3	38 ± 1	80 ± 1
**9**	95 ± 1	24 ± 1	32 ± 2
**10**	73 ± 2	39 ± 1	64 ± 3
**11**	70 ± 3	38 ± 1	75 ± 3
**12**	63 ± 1	84 ± 1	100 ± 3
**13**	140 ± 1	115 ± 2	100 ± 1
**14**	15 ± 0.5	16 ± 0.5	135 ± 9
**15**	105 ± 2	70 ± 2	170 ± 5
**16**	150 ± 3	23 ± 0.5	10 ± 0.5
**17**	83 ± 1	74 ± 1	62 ± 1
**18**	165 ± 6	260 ± 3	140 ± 7
**Cisplatin**	3.0 ± 0.1	3.8 ± 0.2	2.2 ± 0.1

^a^ viability of HeLa cell line at 100 µM of compd **7** was 100%.

### 2.3. Quantitative Structure-Activity Relationships (QSARs) of Cytotoxic Activity

QSAR analysis was performed in order to correlate the cytotoxic activity of the 1,1-dioxo-1,4,2-benzodithiazine derivatives with their chemical structure and to determine the most important parameters controlling their pharmacological effects. The optimized structures of tested compounds were sketched using the geometry optimization function of Spartan software (Spartan ’08, Wavefunction, Inc., Irvine, CA, USA) [20]. The electronic, steric, hydrophilic and hydrophobic features of molecules obtained from molecular modelling descriptors (Table 2 and Table 3) have been applied to the statistical calculations. The QSAR models for each cell line were developed separately. Compound **7** was excluded from the data set of QSAR analysis because of very low cytotoxic activity.

The QSAR model generation was done using the Statistica package (v.10, Statsoft, Tulsa, OK, USA) [21], and to search for optimal QSAR models multiple linear regression (MLR) along with stepwise algorithm were employed. In order to improve the statistical performance of the HCT-116 model, compounds **12** and **18** were removed from dataset. Both compounds were identified as outliers based on the initial experimental *versus* predicted activity plot. A much higher difference between experimental and predicted activity in comparison to the rest of compounds, suggests that these compounds possess some additional features explaining their biological activity, that was not covered in the applied descriptor dataset.

**Table 2:** Theoretical molecular parameters of compounds **6**, **8**‒**18**: CPK Area, CPK Volume, PSA, MW, CFD (HBD), CFD (HBA), Angle (i, j, k), Dihedral (i, j, k, l), Distance (i, j), LogP, Hardness. 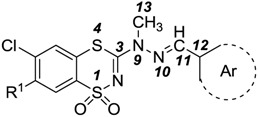

Compd	CPK Area (Å^2^)	CPK Volume (Å^3^)	PSA (Å^2^)	MW (amu)	CFD (HBD)	CFD (HBA)	Angle (C3,N9,N10)	Angle (N9,N10,C11)	Dihedral (C3,N9,N10,C11)	Dihedral (N9,N10,C11,C12)	Distance (S4,C12)	Distance (S1,C12)	LogP	Hardness
**6**	378.32	351.11	89.6827	411.890	2	8	119.59	115.50	69.29	−179.26	6.053	5.332	4.8599	0.199531
**8**	389.58	362.09	69.9921	474.787	1	7	119.89	116.30	65.86	−179.35	6.053	5.325	6.0783	0.197595
**9**	395.72	366.01	109.1826	440.888	1	10	120.39	117.25	61.89	−179.55	6.052	5.320	5.2833	0.201345
**10**	417.97	388.93	74.8555	504.813	1	8	119.77	116.17	66.77	−179.57	6.055	5.335	5.9519	0.195736
**11**	402.80	375.17	68.4649	509.232	1	7	119.73	116.36	65.77	−179.05	6.050	5.317	6.6365	0.197948
**12**	346.63	318.50	63.7233	368.869	1	7	121.27	117.32	59.23	179.13	6.044	5.315	2.9324	0.190920
**13**	370.32	337.44	99.2438	414.850	0	10	120.74	118.17	58.08	−179.47	6.027	5.308	3.6225	0.193076
**14**	377.41	345.96	91.1344	430.917	0	10	121.28	118.67	55.91	179.99	6.045	5.329	4.3443	0.186695
**15**	411.87	380.93	110.6991	455.899	2	9	119.50	115.35	69.58	−179.19	6.053	5.334	4.1929	0.188761
**16**	411.80	380.88	110.6926	455.899	2	9	119.90	116.04	66.55	−179.41	6.058	5.337	4.1929	0.184929
**17**	370.21	345.35	85.0143	406.874	1	8	119.29	115.11	63.61	−177.70	6.017	5.199	4.7956	0.185247
**18**	371.53	338.82	114.5075	425.833	0	11	120.30	117.52	60.87	−179.34	6.027	5.303	3.1687	0.194908

**CPK Area**—surface area of a space-filling model; **CPK Volume**—volume of a space-filling model; **PSA**—polar surface area (N, O + attached hydrogens); **MW**—molecular weight; **CFD (HBD)**—number of hydrogen bond donors; **CFD (HBA)**—number of hydrogen bond acceptors; **Angle (i, j, k)**—angle involving atoms i, j, k (degrees); **Dihedral (i, j, k, l)**—dihedral angle involving atoms i, j, k, l (degrees); **Distance (i, j)**—distance involving atoms i, j (Å); **LogP**—lipophilicity estimated from Crippen model [22]; **Hardness**—-(HOMO—LUMO)/2 (eV).

**Table 3:** Theoretical molecular parameters of compounds **6**, **8**‒**18**: E, E HOMO, E LUMO, Elect (i), Mull (i), Nat (i), Electronegativity.

Compd	E	E HOMO	E LUMO	LUMO-HOMO	Elect (C13)	Mull (C13)	Nat (C13)	Elect (C11)	Mull (C11)	Nat (C11)	Elect (N10)	Mull (N10)	Nat (N10)	Electronegativity
	(kcal/mol)													
**6**	−1462850	−194.26	56.16	250.42	−0.579	−0.303	−0.410	0.330	0.224	0.226	−0.384	−0.352	−0.376	0.110038
**8**	−3029531	−201.90	46.09	247.99	−0.608	−0.306	−0.411	0.299	0.217	0.213	−0.365	−0.338	−0.355	0.124147
**9**	−1543557	−214.66	38.03	252.69	−0.588	−0.308	−0.411	0.253	0.215	0.203	−0.338	−0.338	−0.350	0.140737
**10**	−3100993	−196.45	49.20	245.65	−0.587	−0.306	−0.411	0.297	0.217	0.213	−0.355	−0.337	−0.353	0.117330
**11**	−3317493	−208.48	39.95	248.43	−0.582	−0.307	−0.411	0.301	0.218	0.209	−0.350	−0.334	−0.349	0.134281
**12**	−1355166	−186.14	53.47	239.61	−0.662	−0.306	−0.410	0.175	0.157	0.183	−0.384	−0.327	−0.351	0.105707
**13**	−1495275	−218.12	24.20	242.32	−0.609	−0.311	−0.412	0.143	0.131	0.141	−0.376	−0.297	−0.313	0.154513
**14**	−1697748	−218.76	15.55	234.31	−0.602	−0.311	−0.411	0.189	0.175	0.163	−0.340	−0.301	−0.317	0.161921
**15**	−1580568	−195.25	41.65	236.90	−0.595	−0.304	−0.411	0.350	0.225	0.228	−0.386	−0.351	−0.377	0.122384
**16**	−1580564	−191.14	40.95	232.09	−0.574	−0.307	−0.411	0.347	0.212	0.215	−0.375	−0.334	−0.355	0.119665
**17**	−1448937	−206.78	25.71	232.49	−0.575	−0.307	−0.411	0.326	0.158	0.208	−0.399	−0.310	−0.350	0.144282
**18**	−1528335	−224.84	19.78	244.62	−0.595	−0.313	−0.413	0.150	0.139	0.150	−0.383	−0.296	−0.316	0.163393

**E**—total energy; **E HOMO**—energy of highest-occupied molecular orbital; **E LUMO**—energy of lowest-occupied molecular orbital; **Elect (i)**—electrostatic charge on atom i; **Mull (i)**—Mulliken charge on atom i; **Nat (i)**—natural charge on atom i; **Electronegativity**—-(HOMO + LUMO)/2 (eV).

QSAR models were validated using the leave-one-out cross validation technique. In the case of the quantitative structure-activity relationships for the HeLa cell line a suitable statistical model was not found.

Details of the predictive performance of constructed QSAR models are shown in Table 4. Predicted IC_50_ values were described by equations as the function of significant descriptor values. The observed as well as the predicted cytotoxic activities are given in Table 5. The statistical significance of equations, as well as high R_cv_ value and value of RMSECV comparable to s, suggests that the obtained QSAR model can be used to explain relationships between chemical structure and activity.

**Table 4:** The QSAR equations and their predictive performance in predicting cytotoxic activity of the 1,1-dioxo-1,4,2-benzodithiazine derivatives against MCF-7 and HCT-116 cell lines.

Cell Line	Equation	N	R	s	R_cv_	RMSECV	F	p
MCF-7	IC_50_ = −72361.3 (Nat C13) + 2.8 (E HOMO) − 29088.3	12	0.874	23.52	0.750	28.66	14.592	0.001499
HCT-116	IC_50_ = −45749.1 (Nat C13) − 937.3 (Elect N10) − 19099.8	10	0.902	14.97	0.715	21.54	15.303	0.002782

N—number of compounds in data set; R—a correlation coefficient; s—a standard error of estimate; R_cv_—a correlation coefficient of leave-one-out cross validation (LOO-CV); RMSECV—a root mean square error LOO-CV; F—Fisher test value; p—significance level of F test.

**Table 5:** The cytotoxic activity against MCF-7 and HCT-116 obtained from experiments (observed) and from statistical calculations (predicted).

Compounds	IC_50_ [µM]			
	MCF-7		HCT-116	
	Observed	Predicted	Observed	Predicted
**6**	45 ± 0.5	41	31 ± 1	17
**8**	84 ± 3	92	38 ± 1	45
**9**	95 ± 1	57	24 ± 1	20
**10**	73 ± 2	108	39 ± 1	36
**11**	70 ± 3	74	38 ± 1	31
**12**	63 ± 1	64	84 ± 1	- ^a^
**13**	140 ± 1	120	115 ± 2	101
**14**	15 ± 0.5	46	16 ± 0.5	22
**15**	105 ± 2	111	70 ± 2	65
**16**	150 ± 3	122	23 ± 0.5	54
**17**	83 ± 1	79	74 ± 1	77
**18**	165 ± 6	174	260 ± 3	- ^a^

^a^ compound was identified as a statistical outlier.

**Figure 2:**
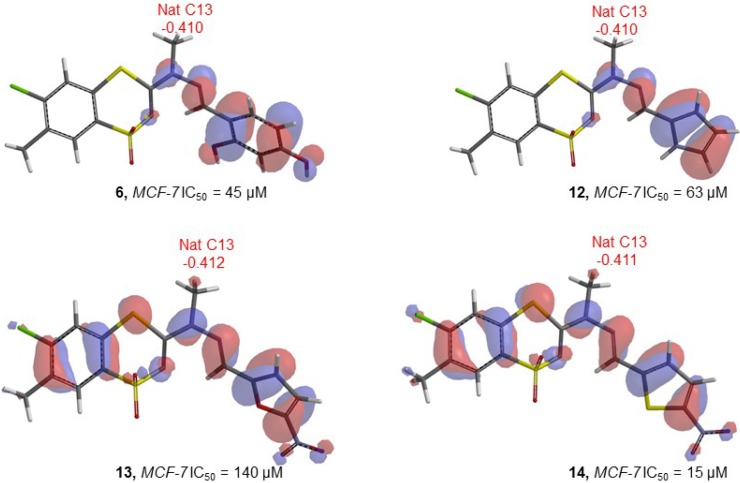
Isosurface plots of HOMO of compounds **6**, **12**, **13** and **14**, and natural charges on atom C13.

The statistical MCF-7 model indicated that natural charge on carbon atom C13 and energy of highest occupied molecular orbital (HOMO) are highly involved in cytotoxic activity against MCF-7 cell line. It was found that natural charge on carbon atom C13 has a negative weight in the correlation, whereas energy of HOMO positively correlates with cytotoxic activity of compounds. The significant influence of electronic properties of atom C13 on biological activity has been illustrated by optimized structures including marked natural charges and isosurface plots of HOMO, that show the energy level of HOMO (Figure 2). The anticancer properties of compounds **6** and **12** have been determined by low natural charge of C13 in spite of low HOMO energy. Apparently differences in charges of C13 had the influence on totally different biological activities of compounds **13** and **14**, despite their similar HOMO energy.

As shown the QSAR model for HCT-116 cell line, natural charge on carbon atom C13 as well as electrostatic charge on nitrogen atom N10 correlates with cytotoxic activity of compound. According to the equation, high anticancer activity is affected by low natural charge of C13 as the most important descriptor and low electrostatic charge of N10. As indicated in Table 3 inactive compounds exhibited relatively larger natural charges on C13 atom.

The QSAR results indicate that the cytotoxic activities of 1,1-dioxo-1,4,2-benzodithiazines against MCF-7 and HCT-116 are related to their molecular structure and especially the nature of the *Ar* group. Taken together the SAR and QSAR results on the cytotoxic activities of these analogs may provide valuable information for the further design of novel anticancer agents.

## 3. Experimental Section

### 3.1. General Information

The melting points were determined on a Boethius PHMK apparatus and are uncorrected. Infrared (IR) spectra were recorded on a Thermo Mattson Satellite FTIR spectrophotometer. The NMR spectra were recorded on a Varian Gemini 200 spectrometer at 200 MHz (^1^H-NMR) or on a Varian Unity 500 Plus apparatus at 500 MHz (^1^H-NMR) and 125 MHz (^13^C-NMR). Chemical shifts are expressed as δ values in parts per million (ppm) relative to TMS as an internal standard. Spectra were acquired in deuterated dimethyl sulfoxide (DMSO-*d_6_*). Elemental analyses were performed on PerkinElmer 2400 Series II CHN Elemental Analyzer and were in agreement with the theoretical values within ±0.4% range. The commercially unavailable new substrates were obtained according to the following methods described previously: **1a** [23], **1b** [24] and **1c** [25]. 

### 3.2. Chemistry

#### 3.2.1. Procedure for the Preparation of *N*-Methyl-*N*-(6-chloro-7-R^1^-1,1-dioxo-1,4,2-benzodithiazin-3-yl)hydrazines **2**–**4**

A mixture of methylhydrazine (2.39 g, 0.052 mol) and the corresponding 3-methylthio-1,4,2-benzodithiazine **1a**–**c** (0.05 mol) in dry acetonitrile (60 mL) was stirred at room temperature for 8 h, followed by reflux until the evolution of MeSH had ceased (10–16 h) (*Caution*: because of its high toxicity, MeSH should be trapped in aqueous NaOH solution). After cooling to room temperature the reaction mixture was stirred for 4 h. The precipitate of the benzodithiazinyl hydrazines obtained was filtered off, washed with acetonitrile (2 × 5 mL) and dried. In this manner the following hydrazines were obtained.

*N-Methyl-N-(6-chloro-7-methyl-1,1-dioxo-1,4,2-benzodithiazin-3-yl)hydrazine* (**2**). Starting from 6-chloro-7-methyl-3-methylthio-1,1-dioxo-1,4,2-benzoditiazine **1a** (14.7 g), the title compound **2** was obtained (13.5 g, 93%): mp 271–272 °C dec.; IR (KBr) ν_max_ 3235 (N-NH_2_), 1645 (C=N), 1345, 1155 (SO_2_) cm^−1^; ^1^H-NMR (500 MHz, DMSO-*d_6_*) δ 2.40 (s, 3H, CH_3_-7), 3.31 (s, 3H, N-CH_3_), 5.70 (s, 2H, N-NH_2_), 7.86 (s, 1H, H-5), 7.92 (s, 1H, H-8) ppm; ^13^C-NMR (125 MHz, DMSO-*d_6_*) δ 19.95, 41.37, 126.66, 128.48, 130.56, 130.59, 137.40, 137.43, 166.82 ppm; anal. C 37.12, H 3.58, N 14.44% calcd for C_9_H_10_ClN_3_O_2_S_2_, C 37.04, H 3.45, N 14.40%.

*Methyl 6-chloro-3-(1-methylhydrazino)-1,1-dioxo-1,4,2-benzodithiazine-7-carboxylate* (**3**). Starting from 6-chloro-3-methylthio-1,1-dioxo-1,4,2-benzodithiazine-7-carboxylate **1b** (16 g), the title compound **3** was obtained (15.1 g, 90%): mp 252–253 °C dec.; IR (KBr) ν_max_ 3360 (N-NH_2_), 1740 (C=O), 1340, 1155 (SO_2_) cm^−1^; ^1^H-NMR (500 MHz, DMSO-*d_6_*) δ 3.30 (s, 3H, N-CH_3_), 3.88 (s, 3H, CH_3_O), 5.78 (s, 2H, N-NH_2_), 8.09 (s, 1H, H-5), 8.29 (s, 1H, H-8) ppm; ^13^C-NMR (125 MHz, DMSO-*d_6_*) δ 41.48, 53.68, 127.17, 129.90, 130.41, 130.88, 135.73, 137.28, 164.25, 166.17 ppm; anal. C 35.72, H 3.10, N 12.57% calcd for C_10_H_10_Cl N_3_O_4_S_2_, C 35.77, H 3.00, N 12.51%.

*N-Methyl-N-(6-chloro-7-cyano-1,1-dioxo-1,4,2-benzodithiazin-3-yl)hydrazine* **(4)**. Starting from 6-chloro-7-cyano-3-methylthio-1,1-dioxo-1,4,2-benzodithiazine **1c** (15.2 g), the title compound **4** was obtained (13.7 g, 91%): mp 285–286 °C dec.; IR (KBr) ν_max_ 3320, 2245 (N-NH_2_), 2235 (C≡N), 1340, 1155 (SO_2_) cm^−1^; ^1^H-NMR (200 MHz, DMSO-*d_6_*) δ 3.35 (s, 3H, N-CH_3_), 5.82 (s, 2H, N-NH_2_), 8.27 (s, 1H, H-5), 8.50 (s, 1H, H-8) ppm; ^13^C-NMR (125 MHz, DMSO-*d_6_*) δ 41.52, 112.64, 115.41, 129.95, 130.53, 130.98, 138.70, 139.43, 165.96 ppm; anal. C 35.70, H 2.40, N 18.52% calcd for C_9_H_7_Cl N_4_O_2_S_2_, C 35.70, H 2.33, N 18.50%.

#### 3.2.2. General Procedure for the Preparation of 6-Chloro-7-methyl-3-(2-arylmethylene-1-methyl-hydrazino)-1,1-dioxo-1,4,2-benzodithiazines **5**–**14**

A mixture of the benzodithiazinyl hydrazine **2** (1.17 g, 4 mmol), the appropriate aryl carbaldehyde (5 mmol) and glacial acetic acid (0.5 mL, catalytic amount) in ethanol (25 mL) was stirred at room temperature for 3 h, followed at reflux for 25 h. After cooling to room temperature and standing overnight, the precipitate was filtered off, washed with ethanol (4 × 2 mL) and dried. In this manner the following benzodithiazines were obtained.

*6-Chloro-7-methyl-3-[2-(hydroxybenzylidene)-1-methylhydrazino]-1,1-dioxo-1,4,2-benzo-dithiazine)* (**5**). Starting from 2-hydroxybenzaldehyde (0.61 g), the title compound **5** was obtained (1.45 g, 92%): mp 313–315 °C dec.; IR (KBr) ν_max_ 3225 (OH), 1340, 1160 (SO_2_) cm^−1^; ^1^H-NMR (200 MHz, DMSO-*d_6_*) δ 2.44 (s, 3H, CH_3_-7), 3.66 (s, 3H, N-CH_3_), 6.91–7.00 (m, 2H, Ph), 7.31–7.40 (m, 1H, Ph), 7.85 (dd, *J*_ortho_ = 7.7 Hz, *J*_meta_ = 1.6 Hz, 1H, H-3, Ph), 8.03 (s, 2H, H-5 and H-8, benzodithiazine), 8.45 (s, 1H, N=CH), 10.35 (br.s, 1H, OH) ppm; anal. C 48.60, H 3.61, N 10.60% calcd for C_16_H_14_ClN_3_O_3_S_2_, C 48.54, H 3.56, N 10.61%.

*6-Chloro-7-methyl-3-[2-(2,4-dihydroxybenzylidene-1-methylhydrazino]-1,1-dioxo-1,4,2-benzodithiazine* (**6**). Starting from 2,4-dihydroxybenzaldehyde (0.69 g), the title compound **6** was obtained (1.62 g, 98%): mp 318–319 °C dec.; IR (KBr) ν_max_ 3395, 3310 (OH), 1630 (C=N), 1340, 1310, 1165, 1150 (SO_2_) cm^−1^; ^1^H-NMR (200 MHz, DMSO-*d_6_*) δ 2.45 (s, 3H, CH_3_-7), 3.65 (s, 3H, N-CH_3_), 6.38–6.45 (m, 2H, H-3 and H-5, Ph), 7.70 (d, *J* = 9.1 Hz, 1H, H-6, Ph), 8.01 (s, 1H, H-5, benzodithiazine), 8.03 (s, 1H, H-8, benzodithiazine), 8.37 (s, 1H, N=CH), 10.10 (s, 1H, OH-4, Ph), 10.24 (s, 1H, OH-2, Ph) ppm.; ^13^C-NMR (125 MHz, DMSO-*d_6_*) δ 19.98, 32.73, 103.16, 109.05, 111.67, 126.93, 128.70, 128.90, 129.82, 130.50, 137.87, 138.11, 143.83, 159.78, 162.62, 164.53 ppm; anal. C 46.60, H 3.50, N 10.19% calcd for C_16_H_14_ClN_3_O_4_S_2_, C 46.65 H, 3.42 N, 10.20%.

*6-Chloro-7-methyl-3[2-(2,5-dihydroxybenzylidene)-1-methylhydrazino]-1,1-dioxo-1,4,2-benzodithiazine* (**7**). Starting from 2,5-dihydroxybenzaldehyde (0.69 g) the title compound **7** was obtained (1.60 g, 97%): mp 314–315 °C dec.; IR (KBr) ν_max_ 3485 (OH), 3385 (OH), 1615 (C=N), 1345, 1305, 1160 (SO_2_) cm^−1^; ^1^H-NMR (200 MHz, DMSO-*d*_6_) δ 2.45 (s, 3H, CH_3_-7), 3.66 (s, 3H, N-CH_3_), 6.80 (s 2H, H-3 and H-4, Ph), 7.23 (s, 1H, H-6, Ph), 7.98 (s, 1H, H-5, benzodithiazine), 8.04 (s, 1H, H-8, benzodithiazine), 8.39 (s, 1H, N=CH), 9.06 (s, 1H, OH-5, Ph), 9.63 (s, 1H, OH-2, Ph) ppm; ^13^C-NMR (125 MHz, DMSO-*d_6_*) δ 20.01, 32.71, 111.95, 118.09, 120.12, 121.27, 126.95, 128.58, 129.59, 130.06, 138.10, 138.38, 143.12, 150.64, 151.04, 165.13 ppm; anal. C 45.66, H 3.51, N 10.28% calcd for C_16_H_14_Cl N_3_O_4_S_2_, C 46.65, H 3.42, N 10.20%.

*6-Chloro-7-methyl-3-[2-(5-bromo-2-hydroxybenzylidene)-1-methylhydrazino]-1,1-dioxo-1,4,2-benzodithiazine* (**8**). Starting from 5-bromo-2-hydroxybenzaldehyde (1.0 g), the title compound **8** was obtained (1.75 g, 92%): mp 330–331 °C dec.; IR (KBr) ν_max_ 3235 (OH), 1610 (C=N), 1335, 1315, 1160 (SO_2_) cm^−1^; ^1^H-NMR (200 MHz, DMSO-*d_6_*) δ 2.44 (s, 3H, CH_3_-7), 3.66 (s, 3H, N-CH_3_), 6.94 (d, *J* = 8.7 Hz, 1H, H-3, Ph), 7.50 (dd, *J*_ortho_ = 8.7 Hz, *J*_meta_ = 2.5 Hz, 1H, H-4, Ph), 7.88 (d, *J*_meta_ = 2.5 Hz, 1H, H-8, Ph), 8.03 (s, 1H, H-5, benzodithiazine), 8.10 (s, 1H, H-8, benzodithiazine), 8.35 (s, 1H, N=CH), 10.70 (s, 1H, OH) ppm; anal. C 40.45, H 2.80, N 8.90% calcd for C_16_H_13_BrClN_3_O_3_S_2_, C 40.47, H 2.76, N 8.85%.

*6-Chloro-7-methyl-3-[2-(2-hydroxy-5-nitrobenzylidene)-1-methylhydrazino]-1,1-dioxo-1,4,2-benzo-dithiazine* (**9**). Starting from 2-hydroxy-5-nitrobenzaldehyde (0.84 g), the title compound **9** was obtained (1.68 g, 95%): mp 327–328 °C dec.; IR (KBr) ν_max_ 3420 (OH), 1610 (C=N), 1340, 1320, 1165 (SO_2_) cm^−1^; ^1^H-NMR (200 MHz, DMSO-*d_6_*) δ 2.44 (s, 3H, CH_3_-7), 3.69 (s, 3H, N-CH_3_), 7.15 (d, *J* = 9.1 Hz, 1H, H-3, Ph), 8.04 (s, 2H, H-5 and H-8, benzodithiazine), 8.24 (dd, *J*_ortho_ = 9.1 Hz, *J*_meta_ = 2.9 Hz, 1H, H-4, Ph), 8.40 (s, 1H, N=CH), 8.60 (d, *J*_meta_ = 2.9 Hz, 1H, H-6, Ph), 11.95 (s, 1H, OH) ppm; anal. C 43.57, H 3.02, N 12.76% calcd for C_16_H_13_ClN_4_O_5_S_2_, C 43.58, H 2.97, N 12.70%.

*6-Chloro-7-methyl-3-[2-(5-bromo-2-hydroxy-3-methoxybenzylidene)-1-methylhydrazino]-1,1-dioxo-1,4,2-benzodithiazine* (**10**). Starting from 5-bromo-2-hydroxy-5-methoxybenzaldehyde (1.16 g), the title compound **10** was obtained (1.95 g, 97%): mp 311–312 °C dec.; IR (KBr) ν_max_ 3500 (OH), 1610 (C=N), 1350, 1310, 1165 (SO_2_) cm^−1^; ^1^H-NMR (200 MHz, DMSO*-d_6_*) δ 2.44 (s, 3H, CH_3_-7), 3.66 (s, 3H, N-CH_3_), 3.88 (s, 3H, N-CH_3_O), 7.24 (d, *J*_meta_ = 1.8 Hz, 1H, Ph), 7.50 (d, *J*_meta_ = 1.8 Hz, 1H, Ph), 8.03 (s, 1H, H-5, benzodithiazine), 8.09 (s, 1H, H-8, benzodithiazine), 8.38 (s, 1H, N=CH), 10.01 (s, 1H, OH) ppm; anal. C 40.51, H 3.00, N 8.36% calcd for C_17_H_15_BrClN_3_O_4_S_2_, C 40.45, H 2.99, N 8.32%.

*6-Chloro-7-methyl-3-[2-(3-bromo-5-chloro-2-hydroxybenzylidene)-1-methylhydrazino]-1,1-dioxo-1,4,2-benzodithiazine* (**11**). Starting from 3-bromo-5-chloro-2-hydroxybezaldehyde (1.18 g), the title compound **11** was obtained (1.95 g, 93%): mp 337–338 °C dec.; IR (KBr) ν_max_ 3425 (OH), 1605 (C=N), 1335, 1315, 1165 (SO_2_) cm^−1^; ^1^H-NMR (200 MHz, DMSO-*d_6_*) δ 2.45 (s, 3H, CH_3_-7), 3.67 (s, 3H, N-CH_3_), 7.72 (d, *J*_meta_ = 2.5 Hz, 1H, Ph), 7.83 (d, *J*_meta_ = 2.5 Hz, 1H, Ph), 8.05 (s, 1H, H-5, benzodithiazine), 8.11 (s, 1H, H-8, benzodithiazine), 8.47 (s, 1H, N=CH), 10.40 (s, 1H, OH) ppm; anal. C 37.78, H 2.40, N 8.26% calcd for C_16_H_12_BrCl_2_N_3_O_3_S_2_, C 37.73, H 2.37, N 8.25%.

*6-Chloro-7-methyl-3-[2-(1H-pyrrol-2-yl)methylene-1-methylhydrazino]-1,1-dioxo-1,4,2-benzodithiazine* (**12**). Starting from 1*H*-pyrrole-2-carbaldehyde (0.48 g), the title compound **12** was obtained (1.30 g, 88%): mp 245–246 °C dec.; IR (KBr) ν_max_ 3420 (NH), 1615 (C=N), 1345, 1310, 1155 (SO_2_) cm^−1^; ^1^H-NMR (200 MHz, DMSO-*d_6_*) δ 2.44 (s, 3H, CH_3_-7), 3.62 (s, 3H, NCH_3_), 6.20–6.25 (m,1H, H-5, pyrrole), 6.69 (t, *J* = 10 Hz, 1H, H-4, pyrrole), 7.10 (s, 1H, H-3, pyrrole), 7.78 (s, 1H, H-5, benzodithiazine), 8.02 (s, 1H, H-8, benzodithiazine), 8.21 (s, 1H, N=CH), 11.46 (s, 1H, NH) ppm; ^13^C-NMR (125 MHz, DMSO-*d_6_*) δ 19.98, 32.95, 110.77, 116.53, 124.83, 126.81, 126.90, 128.35, 130.12, 130.45, 137.87, 138.12, 139.89, 164.40 ppm; anal. C 45.61, H 3.60, N 15.18% calcd for C_14_H_13_ClN_4_O_2_S_2_, C 45.58, H 3.55, N 15.19%.

*6-Chloro-7-methyl-3-[2-(5-nitrofurfurylidene)-1-methylhydrazino]-1,1-dioxo-1,4,2-benzo-dithiazine* (**13**). Starting from 5-nitrofuran-2-carbaldehyde (0.7 g), the title compound **13** was obtained (1.44 g, 87%): mp 281–282 °C dec.; IR (KBr) ν_max_ 1615 (C=N), 1350, 1315, 1165 (SO_2_) cm^−1^; ^1^H-NMR (200 MHz, DMSO-*d_6_*) δ 2.45 (s, 3H, CH_3_-7), 3.65 (s, 3H, NCH_3_), 7.31 (d, *J* = 3.9 Hz, 1H, furan), 7.86 (d, *J* = 3.9 Hz, 1H, furan), 8.04 (s, 1H, H-5, benzodithiazine), 8.06 (s, 1H, H-8, benzodithiazine), 8.38 (s, 1H, N=CH) ppm; ^13^C-NMR (125 MHz, DMSO-*d_6_*) δ 20.01, 33.26, 115.22, 117.70, 127.10, 128.80, 129.12, 129.44, 135.18, 138.41, 138.78, 151.16, 152.93, 165.56 ppm; anal. C 40.55, H 2.68, N 13.55% calcd for C_14_H_11_ClN_4_O_5_S_2_, C 40.53, H 2.67, N 13.50%.

*6-Chloro-7-methyl-3-[2-(5-nitrothiophen-2-ylmethylene)-1-methylhydrazino]-1,1-dioxo-1,4,2-benzo-dithiazine* (**14**). Starting from 5-nitrothiophene-2-carbaldehyde (0.79 g), the title compound **14** was obtained (1.50 g, 87%): mp 335–336 °C dec.; IR (KBr) ν_max_ 1600 (C=N), 1335, 1305, 1160 (SO_2_) cm^−1^; ^1^H-NMR (200 MHz, DMSO-*d_6_*) δ 2.45 (s, 3H, CH_3_-7), 3.65 (s, 3H, NCH_3_), 7.62 (d, *J* = 4.3 Hz, 1H, thiophene), 8.05 (s, 1H, H-5, benzodithiazine), 8.14 (s, 1H, H-8, benzodithiazine), 8.18 (d, *J* = 4.3 Hz, 1H, thiophene), 8.62 (s, 1H, N=CH) ppm; anal. C 39.11, H 2.59, N 13.06% calcd for C_14_H_11_ClN_4_O_4_S_3_, C 39.02, H 2.57, N 13.00%.

#### 3.2.3. Procedure for the Preparation of Methyl 6-Chloro-3-[2-(2,4-dihydroxybenzylidene)-1-methyl-hydrazino]-1,1-dioxo-1,4,2-benzodithiazine-7-carboxylates **15**, **16**

A mixture of methyl 6-chloro-3-(1-methylhydrazino)-1,1-dioxo-1,4,2-benzodithiazine-7-carboxylate **3** (1.01 g, 3 mmol), and the corresponding 2,4- or 2,5-dihydroxbenzaldehyde (0.55 g, 4 mmol) glacial acetic acid (0.4 mL, catalytic amount) and methanol (30 mL) was stirred at room temperature for 4 h, followed at reflux for 28 h. After cooling to room temperature and standing overnight, the precipitate was filtered off, washed with methanol (4 × 3 mL), and dried. In this manner the following benzodithiazines were obtained.

*Methyl 6-chloro-3-[2-(2,4-dihydroxybenzylidene)-1-methylhydrazino]-1,1-dioxo-1,4,2-benzodithiazine-7-carboxylate* (**15**). Starting from 2,4-dihydroxybenzaldehyde, the title compound **15** was obtained (1.30 g, 95%): mp 310–311 °C dec.; IR (KBr) ν_max_ 3320 wide (OH), 1715, (C=O), 1615 (C=N), 1330, 1310, 1150, 1130 (SO_2_) cm^−1^; ^1^H-NMR (200 MHz, DMSO-*d_6_*) δ 3.60 (s, 3H, N-CH_3_), 3.92 (s, 3H, CH_3_O-C=O), 6.37–6.42 (m, 2H, H-5 and H-6, Ph), 7.71(d, *J*_ortho_ = 9.1 Hz, 1H, H-3, Ph), 8.23 (s, 1H, H-5, benzodithiazine), 8.38 (s, 1H, H-8, benzodithiazine), 8.40 (s, 1H, N=CH), 10.15 (s, 1H), 10.25 (s, 1H, OH) ppm; anal. C 44.78, H 3.12, N 9.26% calcd for C_17_H_14_ClN_3_O_6_S_2_, C 44.78, H 3.09, N 9.21%.

*Methyl 6-chloro-3-[2-(2,5-dihydroxybenzylidene)-1-methylhydrazino]-1,1-dioxo-1,4,2-benzodithiazine-7-carboxylate* (**16**). Starting from 2,5-dihydroxybenzaldehyde, the title compound **16** was obtained (1.28 g, 94%): mp 303–304 °C dec.; IR (KBr) ν_max_ 3420, 3310 (OH), 1610 (C=N), 1340, 1305, 1170, 1165 (SO_2_) cm^−1^; ^1^H-NMR (200 MHz, DMSO-*d_6_*) δ 3.68 (s, 3H, N-CH_3_), 3.91 (s, 3H, CH_3_O-C=O), 6.65–6.85 (m, 2H, H-3 and H-4, Ph), 7.23 (d, *J*_meta_ = 1.6 Hz, 1H, H-6, Ph), 8.21 (s, 1H, H-5, benzodithiazine), 8.38 (s, 1H, H-8, benzodithiazine), 8.41 (s, 1H, N=CH), 9.09 (s, 1H, OH), 9.68 (s, 1H, OH) ppm; ^13^C-NMR (125 MHz, DMSO-*d_6_*) δ 32.99, 53.72, 112.00, 118.12, 120.04, 121.50, 127.55, 130.26, 130.61, 131.06, 136.22, 136.25, 143.89, 150.74, 151.24, 164.14, 164.35 ppm; anal. C 44.77, H 3.14, N 9.24% calcd for C_17_H_14_ClN_3_O_6_S_2_, C 44.78, H 3.09, N 9.21%.

#### 3.2.4. Procedure for the Preparation of 6-Chloro-7-cyano-3-(2-arylmethylene-1-methylhydrazino)-1,1-dioxo-1,4,2-benzodithiazines **17**, **18**

A mixture of *N*-methyl-*N*-(6-chloro-7-cyano-1,1-dioxo-1,4,2-benzodithiazine **4**, (0.9 g, 3 mmol) and the corresponding aryl or heteroaryl carbaldehyde (4 mmol), glacial acetic acid (0.5 mL) and 2-methoxyethanol (10 mL) was stirred at room temperature for 2 h, followed at reflux for 9 h. After cooling to room temperature and standing overnight, the precipitate was filtered off, washed with methanol (5 × 2 mL), and dried. In this manner the following benzodithiazines were obtained.

*6-Chloro-7-cyano-3-[2-hydroxybenzylidene)-1-methylhydrazino]-1,1-dioxo-1,4,2-benzodithiazine* (**17**). Starting from 2-hydroxybenzaldehyde (0.49 g), the title compound **17** was obtained (1.10 g, 90%): mp 314–315 °C dec.; IR (KBr) ν_max_ 2235 (C≡N), 1605 (C=N), 1330, 1160 (SO_2_) cm^−1^; ^1^H-NMR (200 MHz, DMSO-*d_6_*) δ 3.69 (s, 3H, N-CH_3_), 6.90–7.00 (m, 2H, Ph), 7.33–7.41 (m, 1H, Ph), 7.84 (d, *J* = 7.9 Hz, 1H, Ph), 8.41 (s, 1H, H-5, benzodithiazine), 8.61 (s, 1H, H-8, benzodithiazine), 8.61 (s, 1H, N=CH), 10.39 (s, 1H, OH) ppm; anal. C 47.20, H 2.79, N 13.85% calcd for C_16_H_11_ClN_4_O_3_S_2_, C 47.23, H 2.72, N 13.77%.

*6-Chloro-7-cyano-3-[2-(5-nitrofurfurylidene)-1-methylhydrazino]-1,1-dioxo-1,4,2-benzodithiazine* (**18**). Starting from 5-nitrofuran-2-carbaldehyde (0.56 g), the title compound **18** was obtained (1.15 g, 90%): mp 302–303 °C dec.; IR (KBr) ν_max_ 2235 (C≡N), 1615 (C=N), 1360, 1325, 1165 (SO_2_) cm^−1^; ^1^H-NMR (DMSO-*d_6_*) δ 3.67 (s, 3H, N-CH_3_), 7.34 ( d, *J* = 3.9 Hz, 1H, furan), 7.86 (d, *J* = 3.9 Hz, 1H, furan), 8.43 (s, 1H, H-5, benzodithiazine), 8.44 (s, 1H, H-8, benzodithiazine), 8.66 (s, 1H, N=CH) ppm; ^13^C NMR (125 MHz, DMSO-*d_6_*) δ 33.60, 113.64, 115.18, 118.30, 130.20, 130.41, 131.35, 136.17, 137.85, 139.37, 150.84, 153.09, 164.56 ppm; anal. C 39.53, H 1.91, N 16.55% calcd for C_14_H_8_ClN_5_O_5_S_2_, C 39.48, H 1.89, N 16.44%.

### 3.3. Cell Culture and Cell Viability Assay

All chemicals, if not stated otherwise, were obtained from Sigma-Aldrich (St. Louis, MO, USA). The MCF-7 cell line was purchased from Cell Lines Services (Eppelheim, Germany), the HeLa and HCT-116 cell lines were obtained from the Department of Microbiology, Tumor and Cell Biology, Karolinska Institute (Stockholm, Sweden). Cells were cultured in in Dulbecco’s modified Eagle’s medium (DMEM) supplemented with 10% fetal bovine serum, 2 mM glutamine, 100 units/mL penicillin, and 100 μg/mL streptomycin. Cultures were maintained in a humidified atmosphere with 5% CO_2_ at 37 °C in an incubator (Heraceus, HeraCell).

Cell viability was determined using the 3-(4,5-dimethylthiazol-2-yl)-2,5-diphenyl-tetrazoliumbromide (MTT) assay. Cells were seeded in 96-well plates at a density of 5 × 10^3^ cells/well and treated for 72 h with the examined compounds in the concentration range 1–100 μM. Cisplatin was used as a control compound and was examined in the concentration range 0.01–10 μM. Following treatment, MTT (0.5 mg/mL) was added to the medium and cells were further incubated for 2 h at 37 °C. Cells were lysed with DMSO and the absorbance of the formazan solution was measured at 550 nm with a plate reader (Victor, 1420 multilabel counter). The optical density of the formazan solution was measured at 550 nm with a plate reader (Victor, 1420 multilabel counter). The experiment was performed in triplicate. Results are expressed as IC_50_ values. Values are expressed as the mean ± SD of at least three independent experiments.

### 3.4. Molecular Modeling Methodology/Calculations

Quantum chemical calculations were carried out to study the molecular geometry and electronic structure of 6-chloro-7-R^1^-3-(2-arylmethylene-1-methylhydrazino)-1,1-dioxo-1,4,2-benzodithiazines using the Spartan '08 Software [20]. The full optimized geometries of compounds **6**‒**18** in vacuum were calculated using *ab-initio* method at the Hartree-Fock (RHF) with 6-31G* polarization basis set.

Multiple linear regression (MLR, along with stepwise algorithm) analysis were performed using Statistica software [21]. The molecular descriptors were used as independent variables. The dependent variable was cytotoxic activity expressed as IC_50_ values.

In the leave-one-out cross-validation procedure, a data point (compound) was removed from the analyzed set, the regression was recalculated, and then the predicted value for that point was compared to its observed value. This process was repeated until each datum had been omitted once, and then the sum of squares of these errors for the omitted data were used to calculate the cross-validated root-mean-square error (RMSECV). The RMSECV was calculated using following equation:

(1) $$RMSECV=\sqrt{\frac{\sum_{i=1}^{n}{\left(y_{i}-{ŷ}_{i}\right)}^{2}}{N}}$$

where y_i_ is observed activity, ŷ_i_ is predicted activity, N is the sample size of the data set.

## 4. Conclusions

We have developed facile methods for the synthesis of new 6-chloro-7-R^1^-3-(2-arylmethylene-1-methylhydrazino)-1,1-dioxo-1,4,2-benzodithiazines from *N*-methyl-*N*-(6-chloro-7-R^1^-1,1-dioxo-1,4,2-benzodithiazin-3-yl)hydrazines and the appropriate aldehydes. The newly synthesized compounds were tested for their *in vitro* cytotoxic activity against the MCF-7, HCT-116 and HeLa cell lines. The most active compounds **6** and **9** shown the best average cytotoxic activities against all cancer cell lines. Compound **16** exhibited the strongest cytotoxic activity against HeLa cell line with IC_50_ value of 10 µM, while **14** was the most cytotoxic compound against MCF-7 and HCT-116 cell lines, giving the IC_50_ values of 15 µM and 16 µM, respectively. Performed QSAR analysis demonstrate the importance of some electronic properties of molecules in the cytotoxic activities against MCF-7 and HCT-116 cell lines. The QSAR equation showed that a low charge on the C13 carbon atom and high energy of HOMO are likely required for potent cytotoxic activity against MCF-7 cell line. On the other hand, QSAR HCT-116 model indicated that low natural charge on the C13 atom and low electrostatic charge on the N10 atom have an impact on the cytotoxic activity of compounds against the HCT-116 cell line. The QSAR results on the cytotoxic activities of the 1,1-dioxo-1,4,2-benzodithiazine analogs will surely provide useful information for the design of new anticancer agents.

## Supplementary Materials

Supplementary materials can be accessed at: http://www.mdpi.com/1420-3049/20/04/5754/s1.
