# Supplementary material for: Synthesis and QSAR Study of Novel 6-Chloro-3-(2-Arylmethylene-1-methylhydrazino)-1,4,2-benzodithiazine 1,1-Dioxide Derivatives with Anticancer Activity
Source: Molecules. 2015 Apr 1;20(4):5754–70. doi: 10.3390/molecules20045754 (PMC6272346; doi:10.3390/molecules20045754)
Supplement: Supplementary file 1 [file molecules-20-05754-s001.pdf]

Supplementary Materials

Table of Contents

Spectrum 1. ^{1}H-NMR of compd **2** (500 MHz, DMSO-d_6).
Spectrum 2. ^{13}C-NMR of compd **2** (125 MHz, DMSO-d_6).
Spectrum 3. ^{1}H-NMR of compd **3** (500 MHz, DMSO-d_6).
Spectrum 4. ^{13}C-NMR of compd **3** (125 MHz, DMSO-d_6).
Spectrum 5. ^{1}H-NMR of compd **4** (200 MHz, DMSO-d_6).
Spectrum 6. ^{13}C-NMR of compd **4** (125 MHz, DMSO-d_6).
Spectrum 7. ^{1}H-NMR of compd **6** (200 MHz, DMSO-d_6).
Spectrum 8. ^{13}C-NMR of compd **6** (125 MHz, DMSO-d_6).
Spectrum 9. ^{1}H-NMR of compd **7** (200 MHz, DMSO-d_6).
Spectrum 10. ^{13}C-NMR of compd **7** (125 MHz, DMSO-d_6).
Spectrum 11. ^{1}H-NMR of compd **8** (200 MHz, DMSO-d_6).
Spectrum 12. ^{1}H-NMR of compd **11** (200 MHz, DMSO-d_6).
Spectrum 13. ^{1}H-NMR of compd **12** (200 MHz, DMSO-d_6).
Spectrum 14. ^{13}C-NMR of compd **12** (125 MHz, DMSO-d_6).
Spectrum 15. ^{1}H-NMR of compd **13** (200 MHz, DMSO-d_6).
Spectrum 16. ^{13}C-NMR of compd **13** (125 MHz, DMSO-d_6).
Spectrum 17. ^{1}H-NMR of compd **16** (200 MHz, DMSO-d_6).
Spectrum 18. ^{13}C-NMR of compd **16** (125 MHz, DMSO-d_6).
Spectrum 19. ^{1}H-NMR of compd **18** (200 MHz, DMSO-d_6).
Spectrum 20. ^{13}C-NMR of compd **18** (125 MHz, DMSO-d_6).

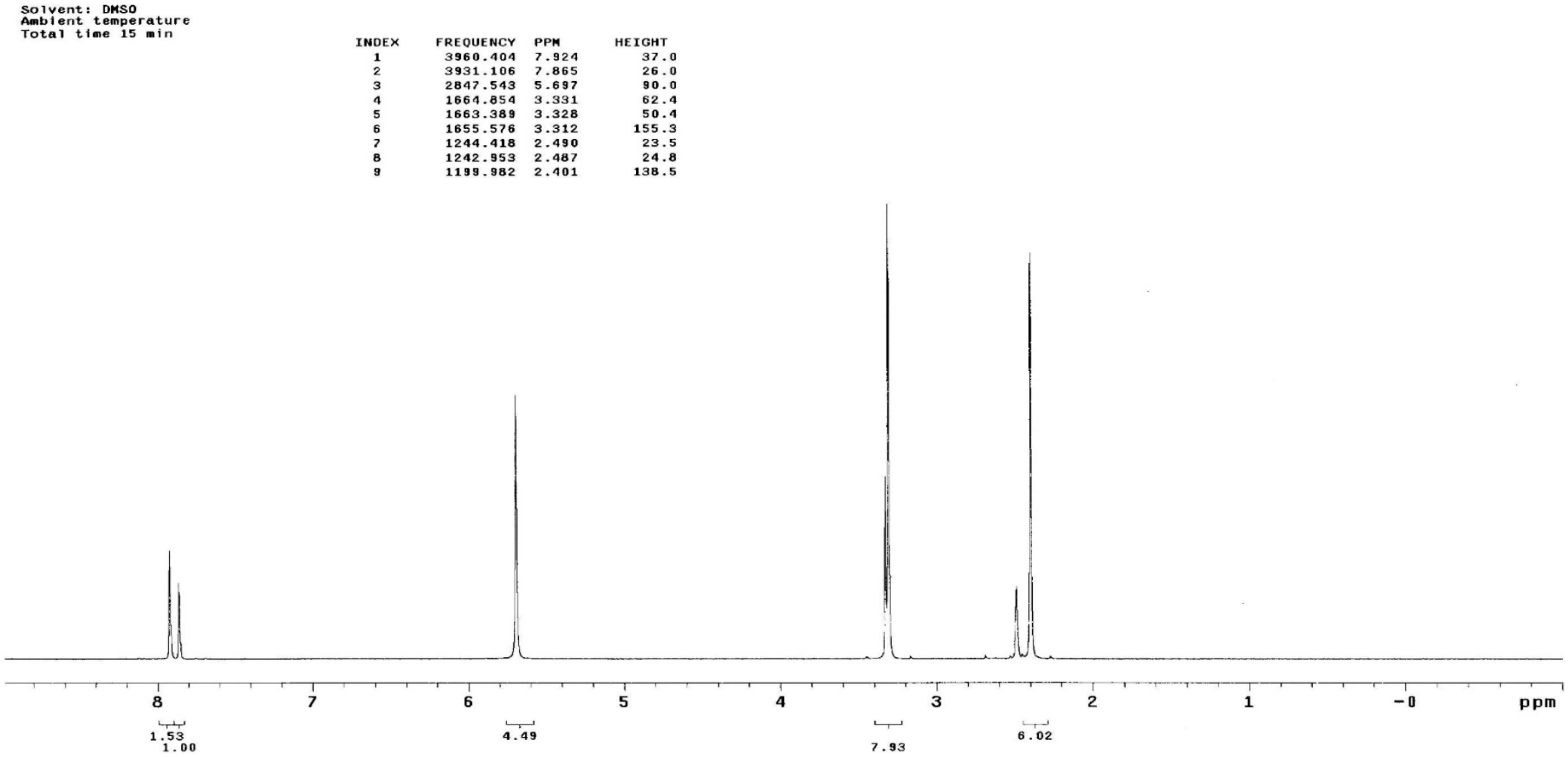

Spectrum 1. ^{1}H-NMR of compd **2** (500 MHz, DMSO-d_6).

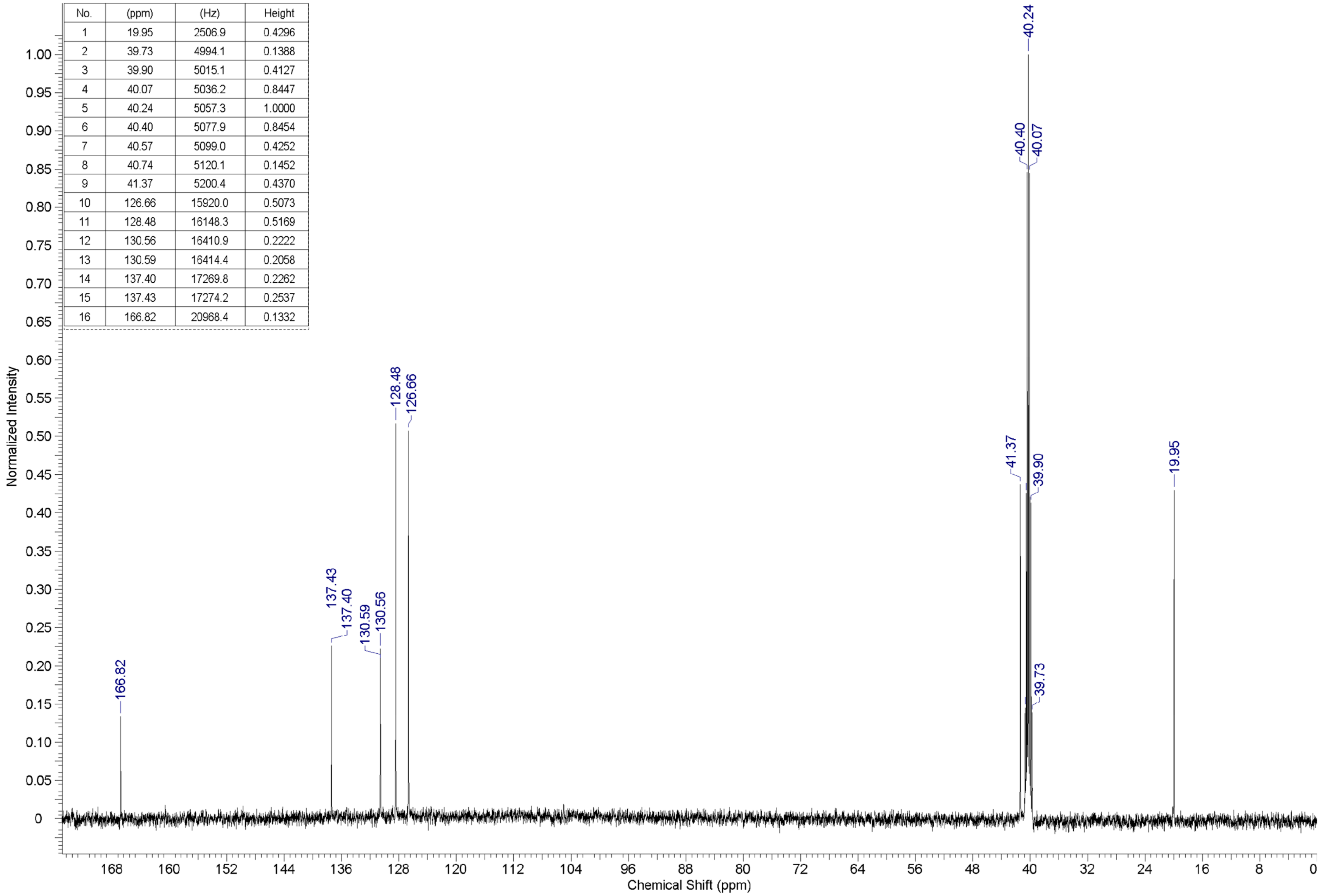

No.	(ppm)	(Hz)	Height
1	19.95	2506.9	0.4296
2	39.73	4994.1	0.1388
3	39.90	5015.1	0.4127
4	40.07	5036.2	0.8447
5	40.24	5057.3	1.0000
6	40.40	5077.9	0.8454
7	40.57	5099.0	0.4252
8	40.74	5120.1	0.1452
9	41.37	5200.4	0.4370
10	126.66	15920.0	0.5073
11	128.48	16148.3	0.5169
12	130.56	16410.9	0.2222
13	130.59	16414.4	0.2058
14	137.40	17269.8	0.2262
15	137.43	17274.2	0.2537
16	166.82	20968.4	0.1332

Spectrum 2. ^{13}C-NMR of compd **2** (125 MHz, DMSO-d_6).

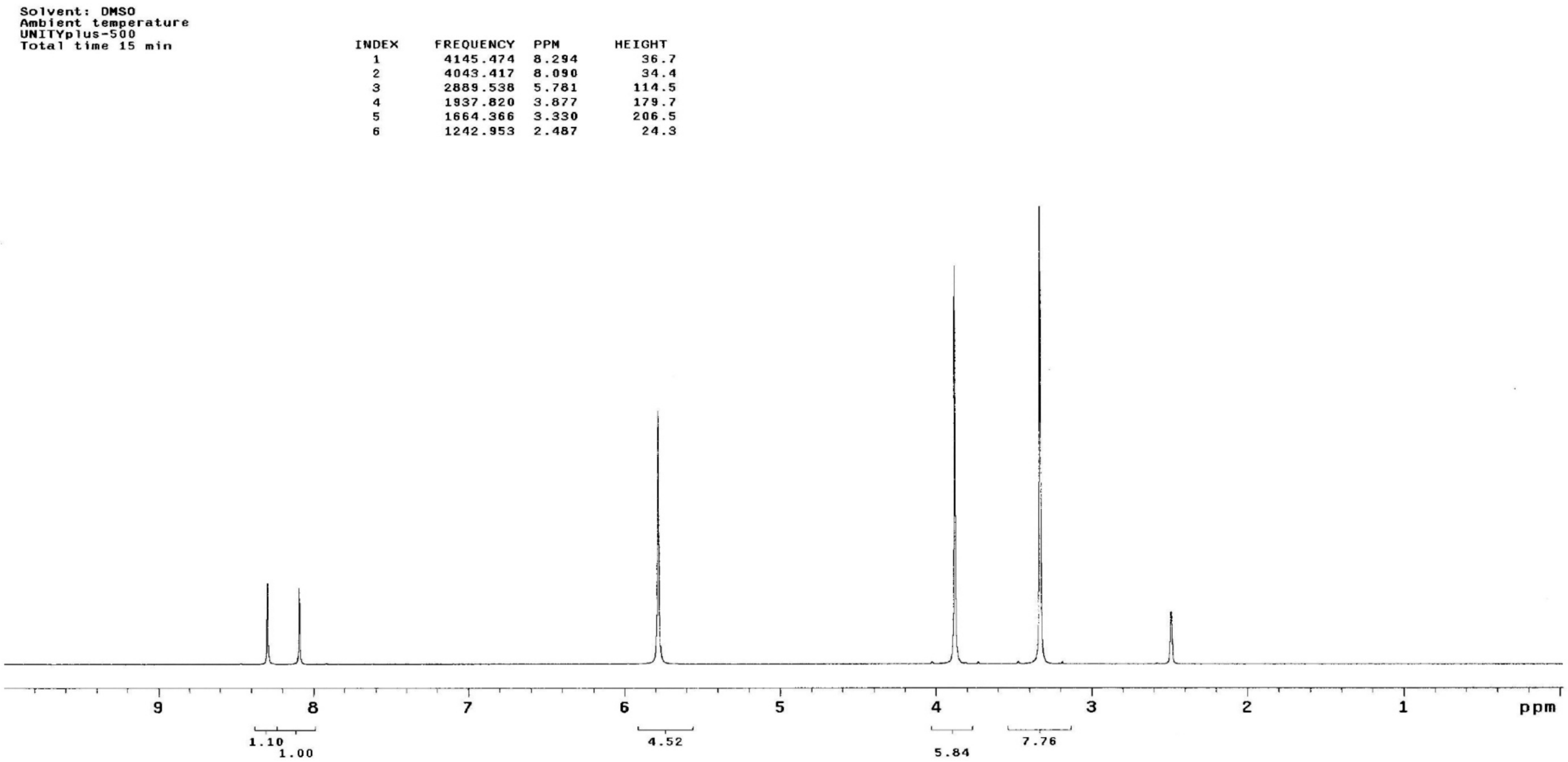

Spectrum 3. ^{1}H-NMR of compd **3** (500 MHz, DMSO-d_6).

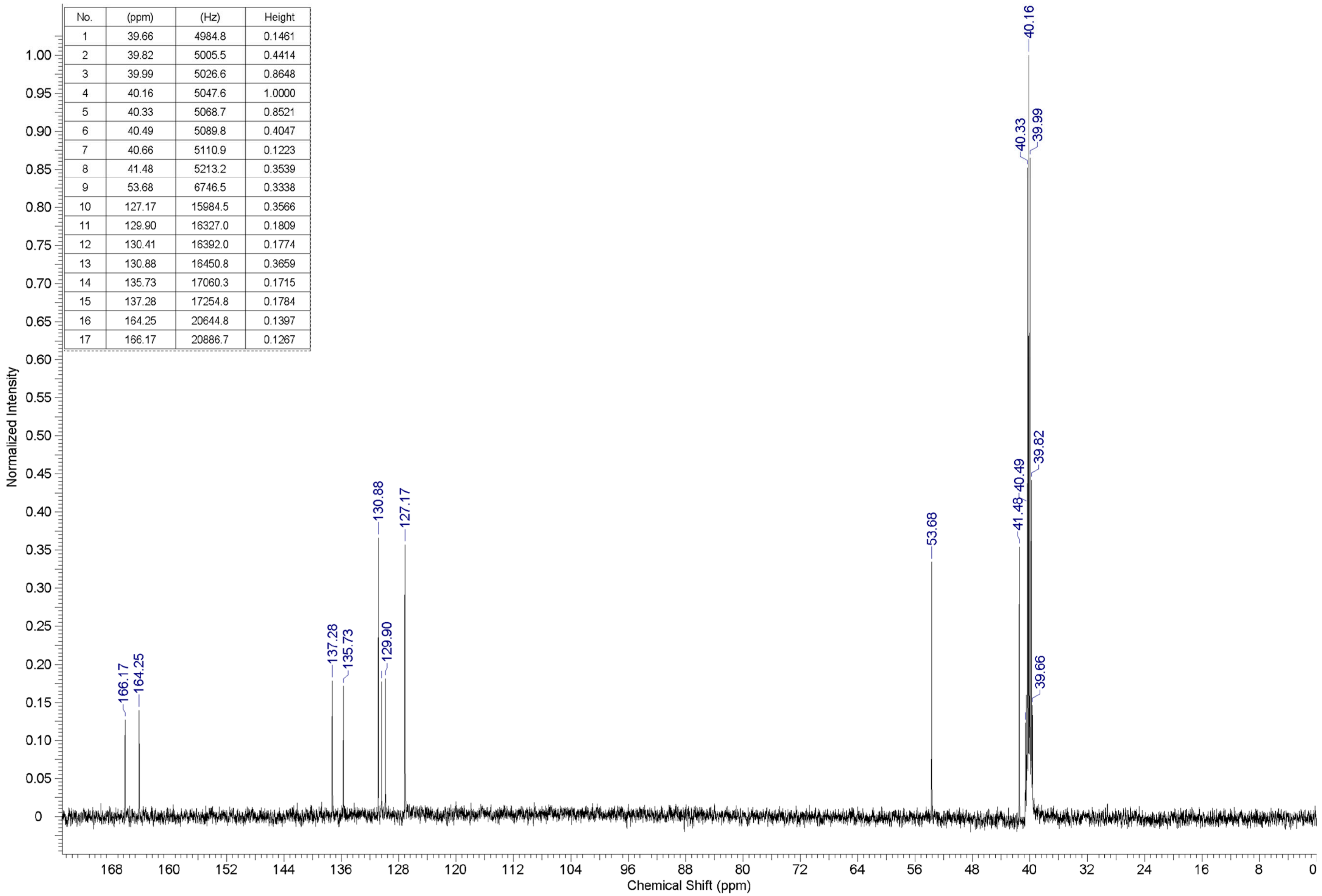

No.	(ppm)	(Hz)	Height
1	39.66	4984.8	0.1461
2	39.82	5005.5	0.4414
3	39.99	5026.6	0.8648
4	40.16	5047.6	1.0000
5	40.33	5068.7	0.8521
6	40.49	5089.8	0.4047
7	40.66	5110.9	0.1223
8	41.48	5213.2	0.3539
9	53.68	6746.5	0.3338
10	127.17	15984.5	0.3566
11	129.90	16327.0	0.1809
12	130.41	16392.0	0.1774
13	130.88	16450.8	0.3659
14	135.73	17060.3	0.1715
15	137.28	17254.8	0.1784
16	164.25	20644.8	0.1397
17	166.17	20886.7	0.1267

Spectrum 4. ^{13}C-NMR of compd **3** (125 MHz, DMSO-*d*$_6$).

SPECTRAL LINES FOR TH= 4.35
RFL= 560.1 RFP= 499.9

INDEX	FREQ	PPM	INTENSIT
01	1700.16	8.502	59.14
02	1655.10	8.277	59.54
03	1164.49	5.828	70.25
04	669.90	3.350	180.21
05	666.72	3.334	137.87
06	504.25	2.522	14.96
07	502.42	2.512	31.26
08	500.63	2.503	42.66
09	498.80	2.494	30.75
10	496.97	2.485	14.01

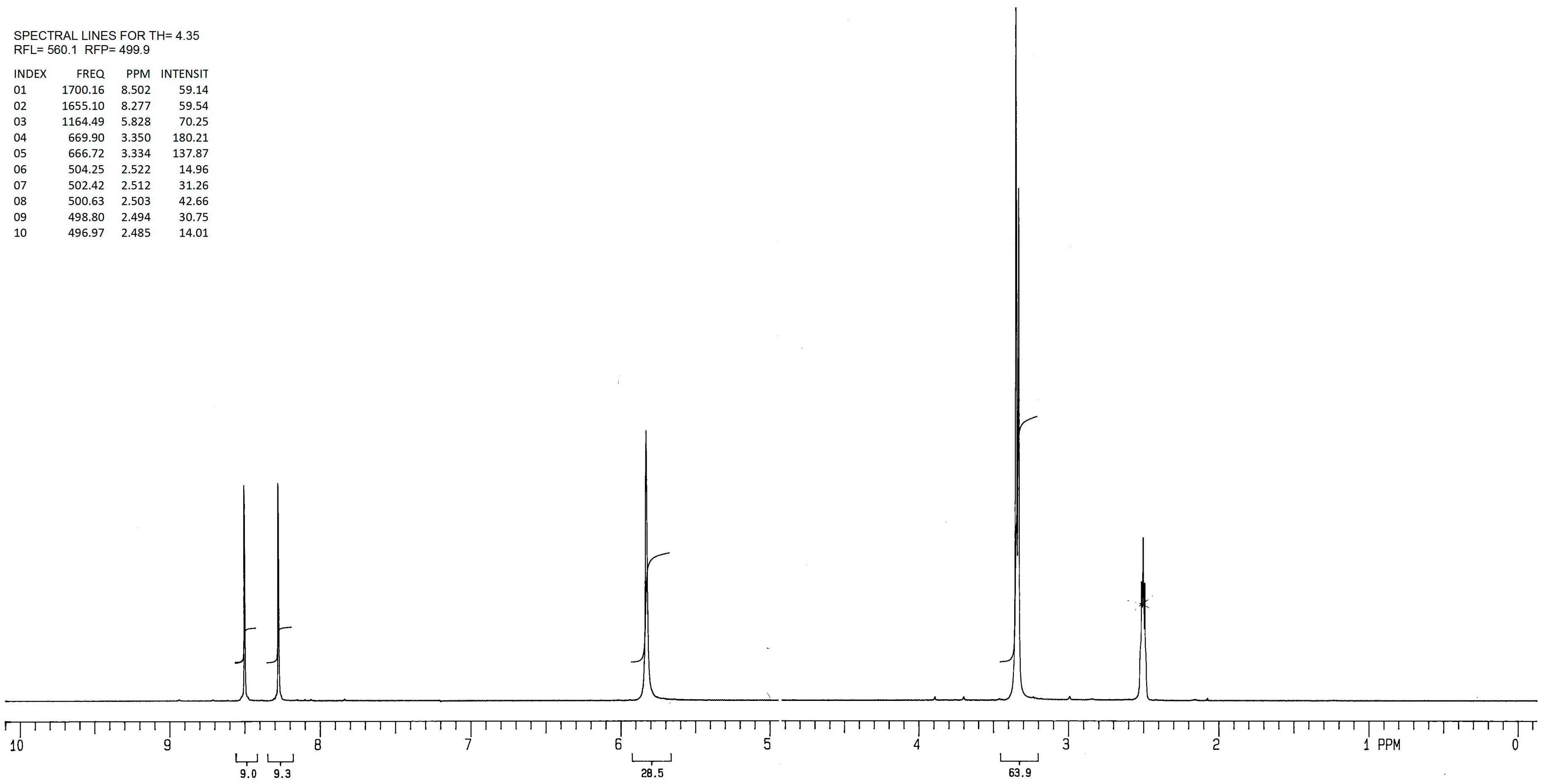

Spectrum 5. ^{1}H-NMR of compd **4** (200 MHz, DMSO-d_6).

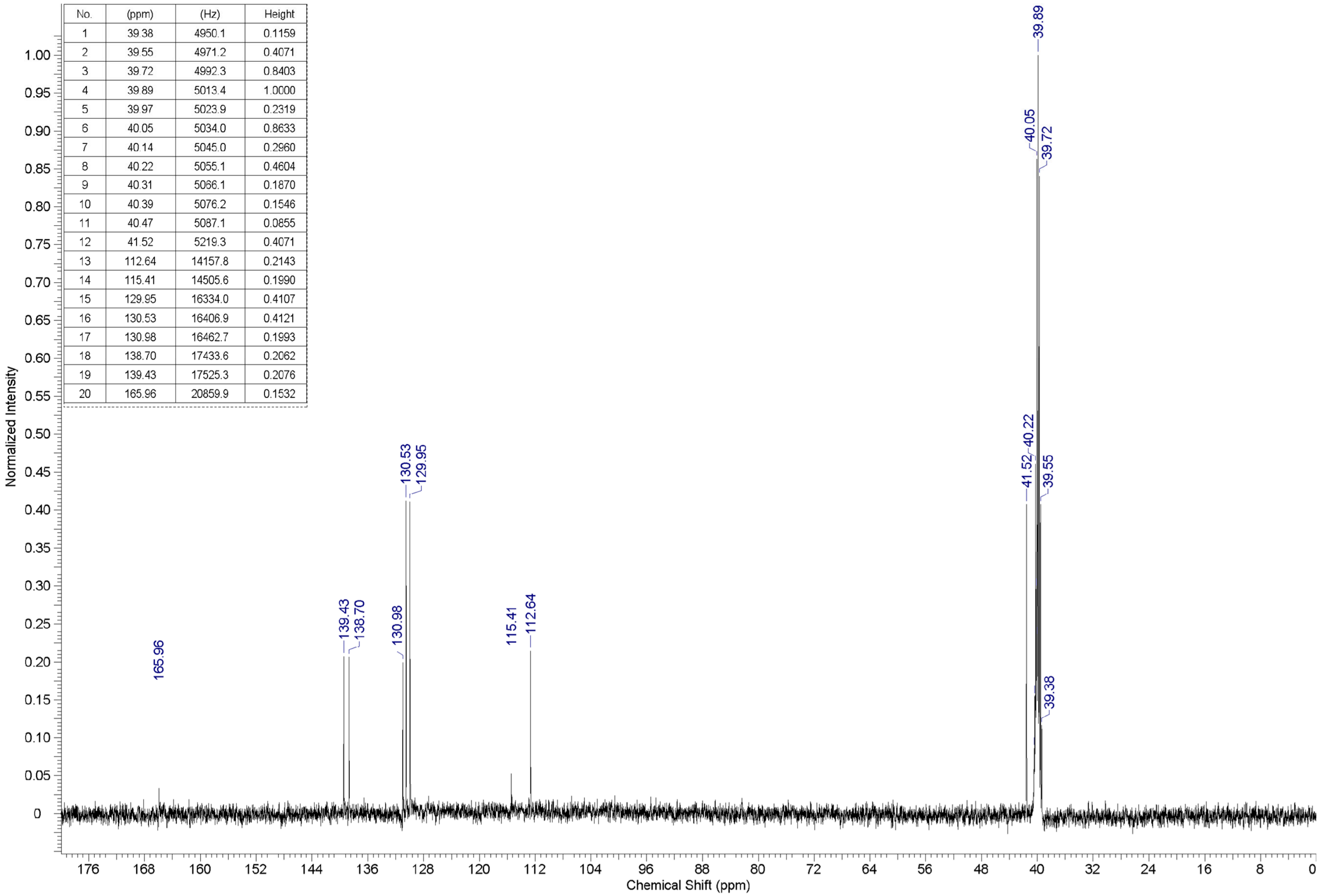

No.	(ppm)	(Hz)	Height
1	39.38	4950.1	0.1159
2	39.55	4971.2	0.4071
3	39.72	4992.3	0.8403
4	39.89	5013.4	1.0000
5	39.97	5023.9	0.2319
6	40.05	5034.0	0.8633
7	40.14	5045.0	0.2960
8	40.22	5055.1	0.4604
9	40.31	5066.1	0.1870
10	40.39	5076.2	0.1546
11	40.47	5087.1	0.0855
12	41.52	5219.3	0.4071
13	112.64	14157.8	0.2143
14	115.41	14505.6	0.1990
15	129.95	16334.0	0.4107
16	130.53	16406.9	0.4121
17	130.98	16462.7	0.1993
18	138.70	17433.6	0.2062
19	139.43	17525.3	0.2076
20	165.96	20859.9	0.1532

Spectrum 6. ^{13}C-NMR of compd **4** (125 MHz, DMSO-d_6).

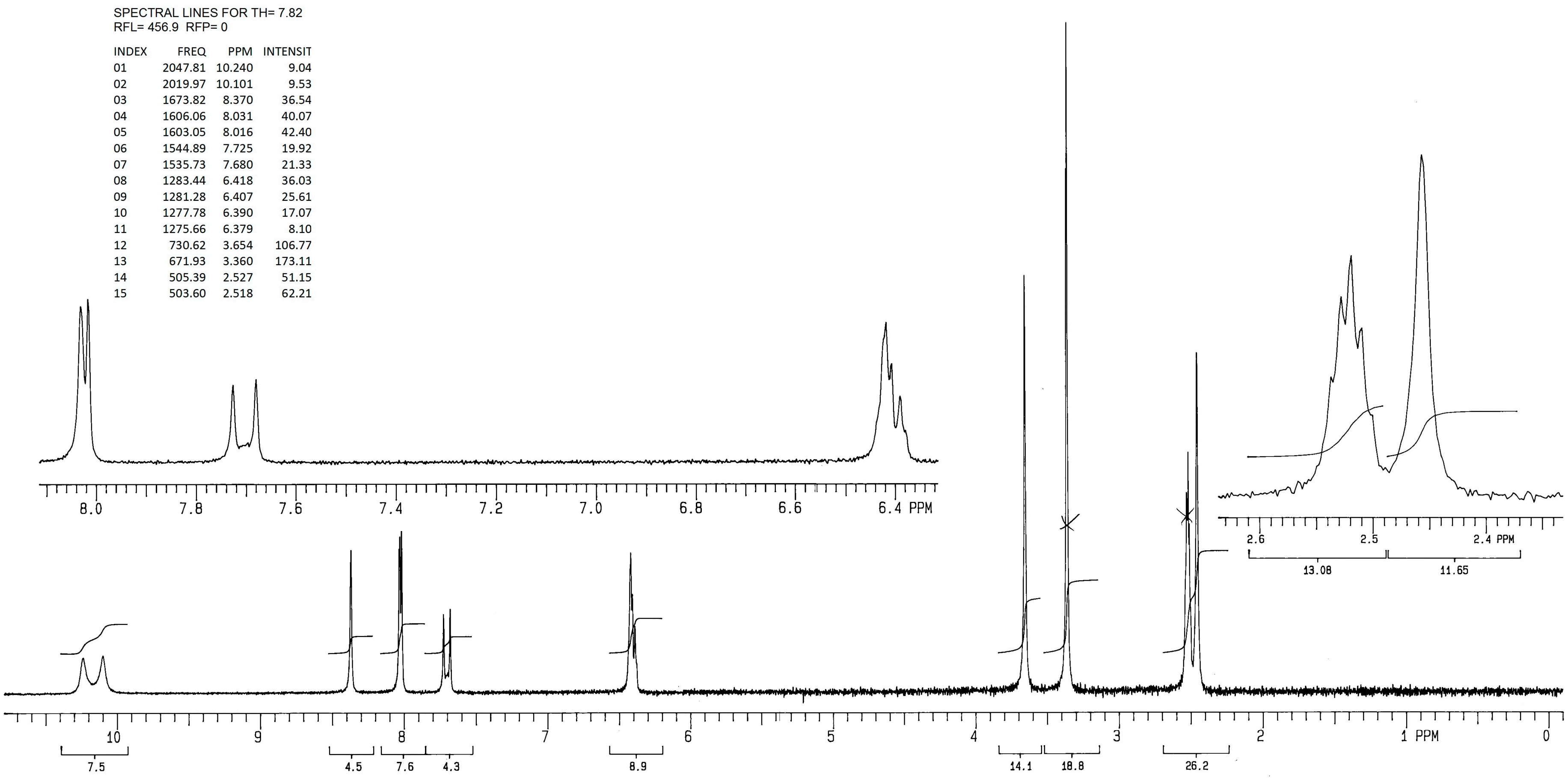

Spectrum 7. ^{1}H-NMR of compd **6** (200 MHz, DMSO-d_6).

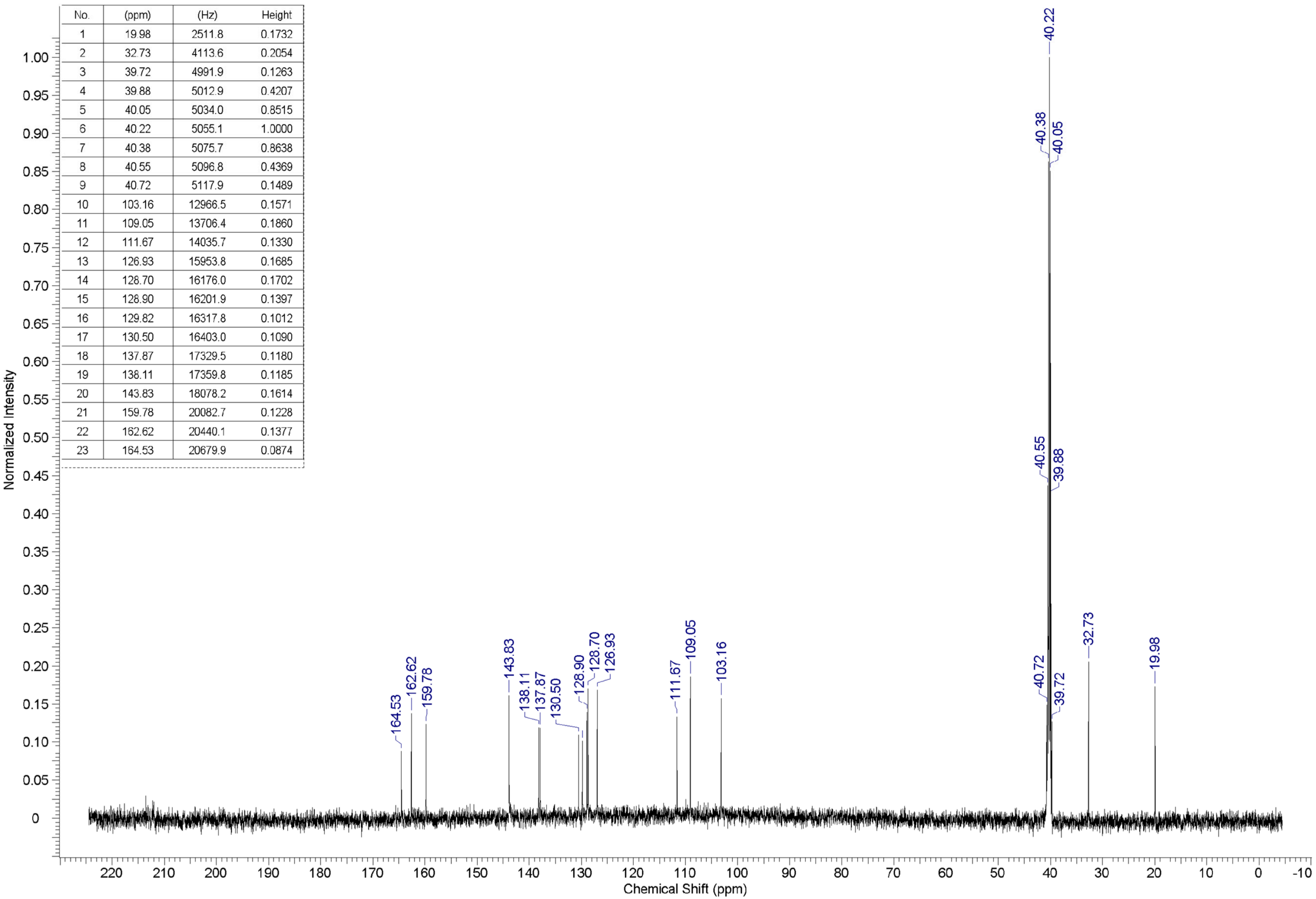

No.	(ppm)	(Hz)	Height
1	19.98	2511.8	0.1732
2	32.73	4113.6	0.2054
3	39.72	4991.9	0.1263
4	39.88	5012.9	0.4207
5	40.05	5034.0	0.8515
6	40.22	5055.1	1.0000
7	40.38	5075.7	0.8638
8	40.55	5096.8	0.4369
9	40.72	5117.9	0.1489
10	103.16	12966.5	0.1571
11	109.05	13706.4	0.1860
12	111.67	14035.7	0.1330
13	126.93	15953.8	0.1685
14	128.70	16176.0	0.1702
15	128.90	16201.9	0.1397
16	129.82	16317.8	0.1012
17	130.50	16403.0	0.1090
18	137.87	17329.5	0.1180
19	138.11	17359.8	0.1185
20	143.83	18078.2	0.1614
21	159.78	20082.7	0.1228
22	162.62	20440.1	0.1377
23	164.53	20679.9	0.0874

Spectrum 8. ^{13}C-NMR of compd **6** (125 MHz, DMSO-d_6).

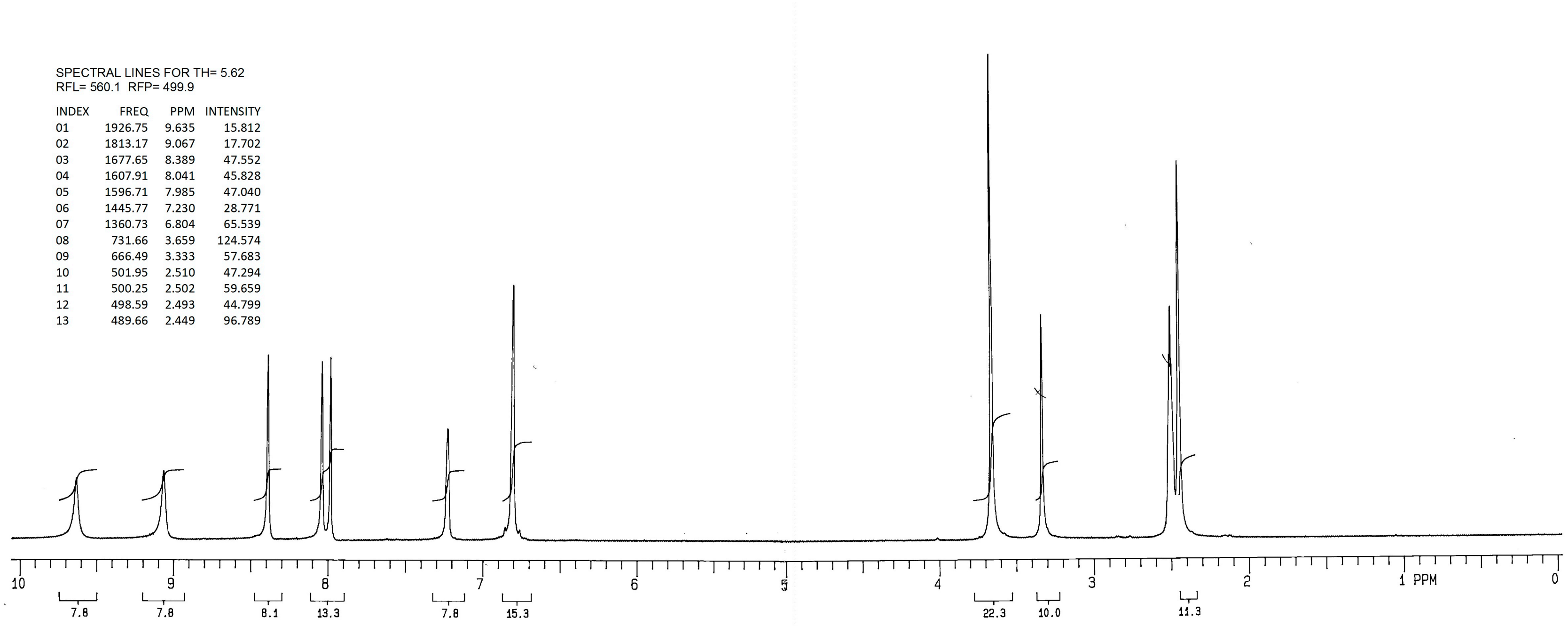

Spectrum 9. ^{1}H-NMR of compd **7** (200 MHz, DMSO-d_6).

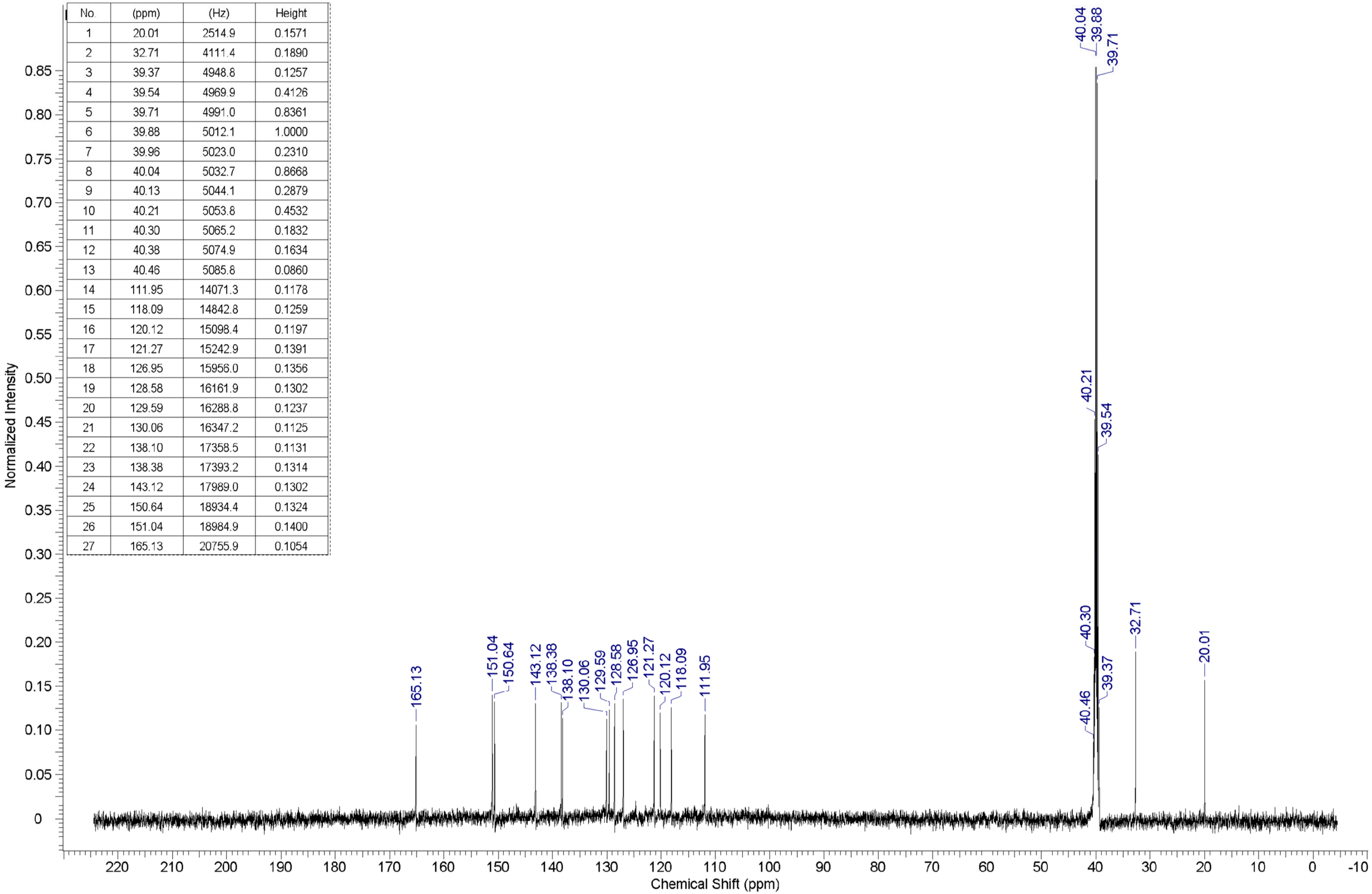

No.	(ppm)	(Hz)	Height
1	20.01	2514.9	0.1571
2	32.71	4111.4	0.1890
3	39.37	4948.8	0.1257
4	39.54	4969.9	0.4126
5	39.71	4991.0	0.8361
6	39.88	5012.1	1.0000
7	39.96	5023.0	0.2310
8	40.04	5032.7	0.8668
9	40.13	5044.1	0.2879
10	40.21	5053.8	0.4532
11	40.30	5065.2	0.1832
12	40.38	5074.9	0.1634
13	40.46	5085.8	0.0860
14	111.95	14071.3	0.1178
15	118.09	14842.8	0.1259
16	120.12	15098.4	0.1197
17	121.27	15242.9	0.1391
18	126.95	15956.0	0.1356
19	128.58	16161.9	0.1302
20	129.59	16288.8	0.1237
21	130.06	16347.2	0.1125
22	138.10	17358.5	0.1131
23	138.38	17393.2	0.1314
24	143.12	17989.0	0.1302
25	150.64	18934.4	0.1324
26	151.04	18984.9	0.1400
27	165.13	20755.9	0.1054

Spectrum 10. ^{13}C-NMR of compd **7** (125 MHz, DMSO-d_6).

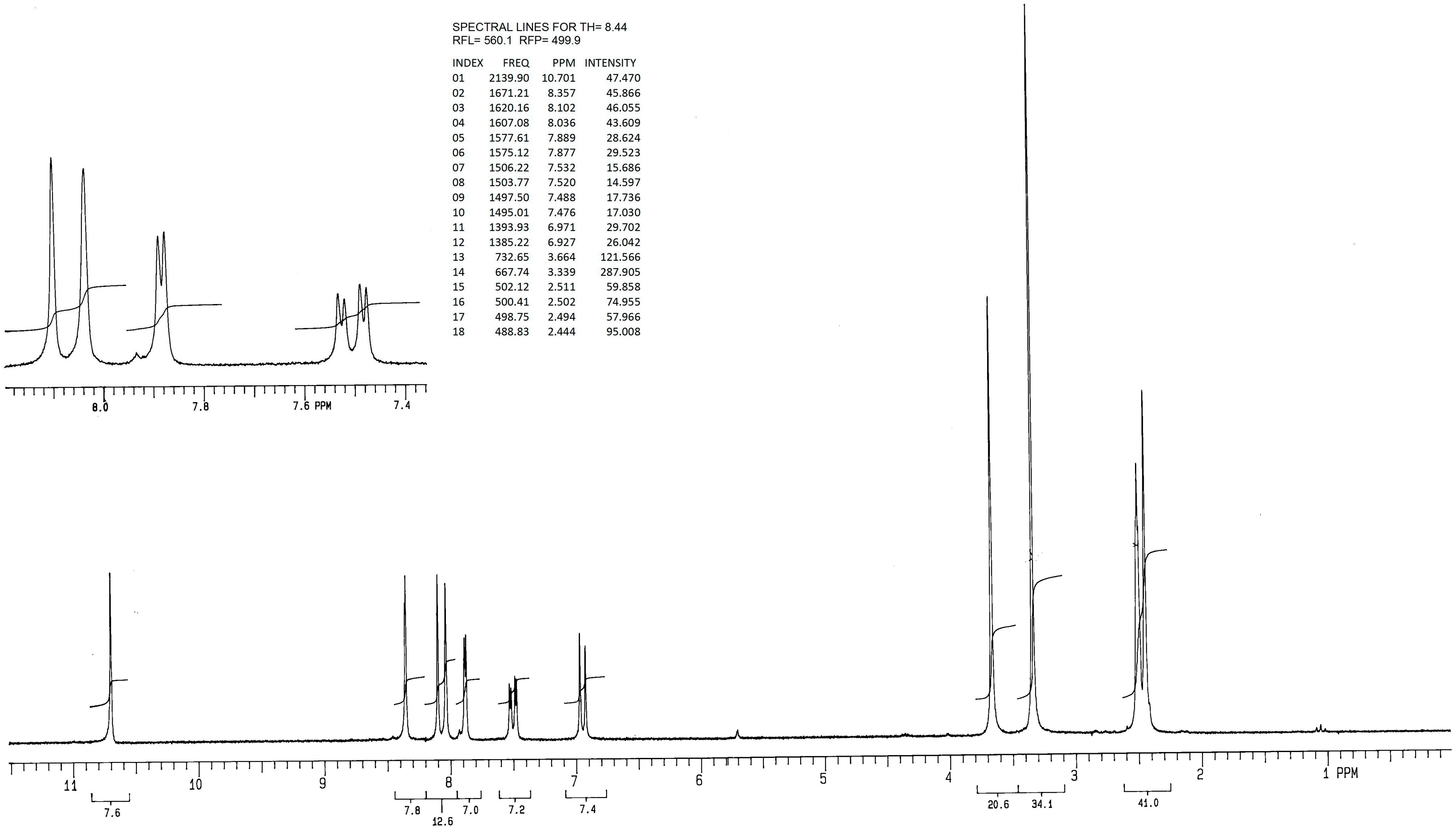

SPECTRAL LINES FOR TH= 8.44
RFL= 560.1 RFP= 499.9

INDEX	FREQ	PPM	INTENSITY
01	2139.90	10.701	47.470
02	1671.21	8.357	45.866
03	1620.16	8.102	46.055
04	1607.08	8.036	43.609
05	1577.61	7.889	28.624
06	1575.12	7.877	29.523
07	1506.22	7.532	15.686
08	1503.77	7.520	14.597
09	1497.50	7.488	17.736
10	1495.01	7.476	17.030
11	1393.93	6.971	29.702
12	1385.22	6.927	26.042
13	732.65	3.664	121.566
14	667.74	3.339	287.905
15	502.12	2.511	59.858
16	500.41	2.502	74.955
17	498.75	2.494	57.966
18	488.83	2.444	95.008

Spectrum 11. ^{1}H-NMR of compd **8** (200 MHz, DMSO-d_6).

SPECTRAL LINES FOR TH= 5.62
RFL= 560.1 RFP= 499.9

INDEX	FREQ	PPM	INTENSITY
01	1694.25	8.472	25.027
02	1622.69	8.114	26.467
03	1610.20	8.052	25.218
04	1568.48	7.843	15.858
05	1565.94	7.831	21.100
06	1555.04	7.776	22.064
07	1552.50	7.763	15.933
08	735.19	3.676	72.962
09	672.05	3.361	6.487
10	668.93	3.345	7.346
11	664.88	3.325	8.182
12	662.75	3.314	7.659
13	661.67	3.309	7.189
14	660.22	3.302	6.870
15	657.98	3.290	5.975
16	657.10	3.286	5.810
17	502.16	2.511	71.090
18	500.41	2.502	90.884
19	498.63	2.493	67.944
20	490.28	2.452	57.538

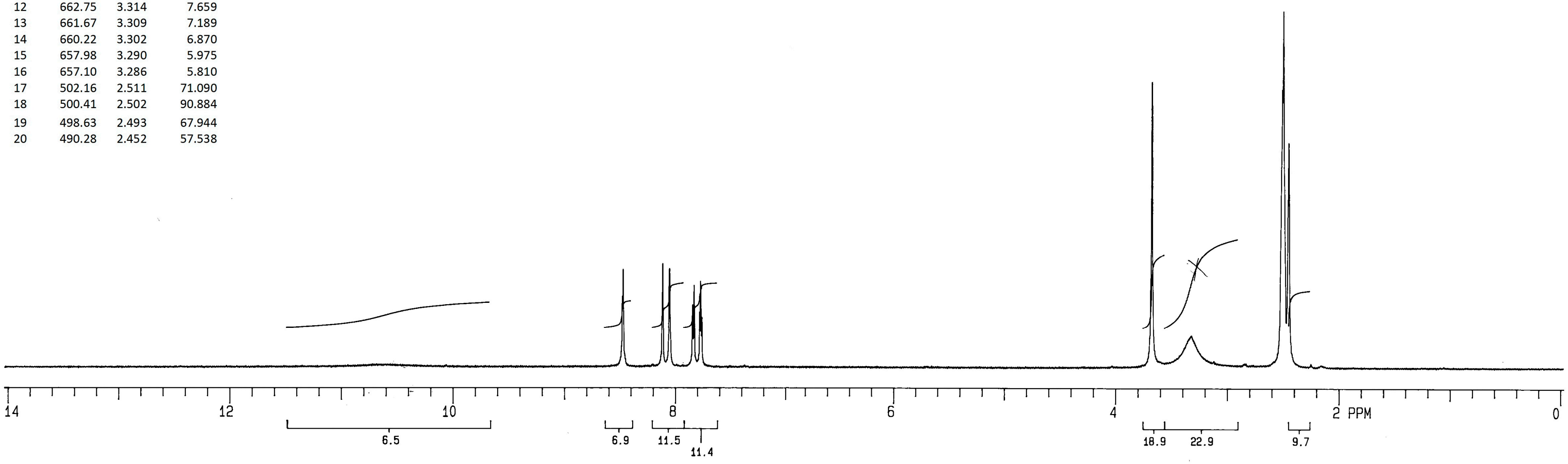

Spectrum 12. ^{1}H-NMR of compd **11** (200 MHz, DMSO-d_6).

SPECTRAL LINES FOR TH= 5.65
RFL= 560.1 RFP= 499.9

INDEX	FREQ	PPM	INTENSITY
01	2296.92	11.486	12.661
02	2295.46	11.479	11.774
03	2292.81	11.465	7.920
04	2291.77	11.460	6.344
05	1641.33	8.208	46.940
06	1605.01	8.026	43.145
07	1586.90	7.936	10.999
08	1574.29	7.872	9.714
09	1555.15	7.777	39.496
10	1419.83	7.100	23.470
11	1339.81	6.700	21.082
12	1338.36	6.693	19.388
13	1337.85	6.690	18.095
14	1250.06	6.251	20.168
15	1248.61	6.244	18.766
16	1247.66	6.239	19.490
17	1245.13	6.226	10.028
18	724.60	3.623	131.462
19	667.69	3.339	300.240
20	500.41	2.502	75.583
21	489.04	2.446	103.730

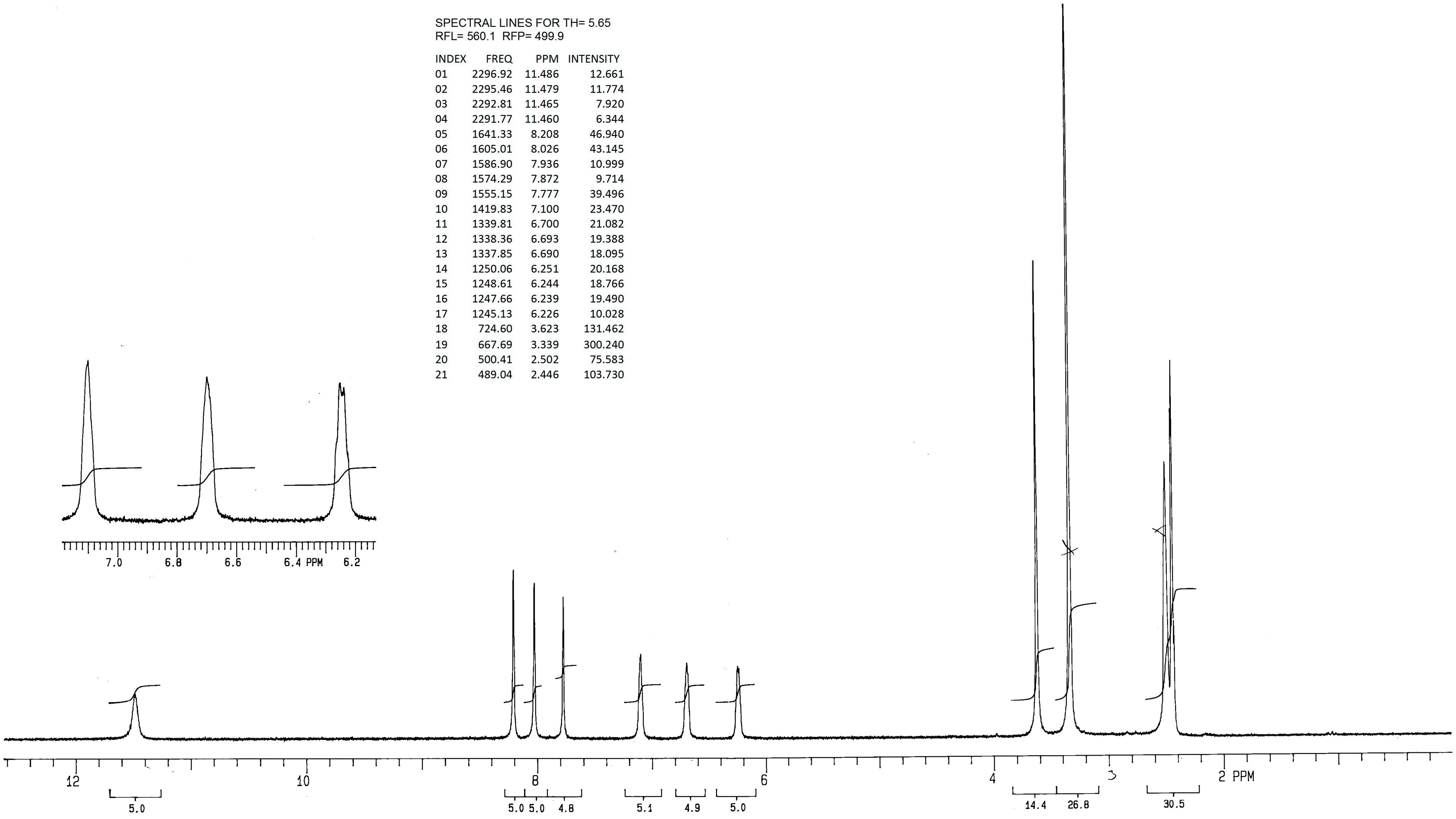

Spectrum 13. ^{1}H-NMR of compd **12** (200 MHz, DMSO-d_6).

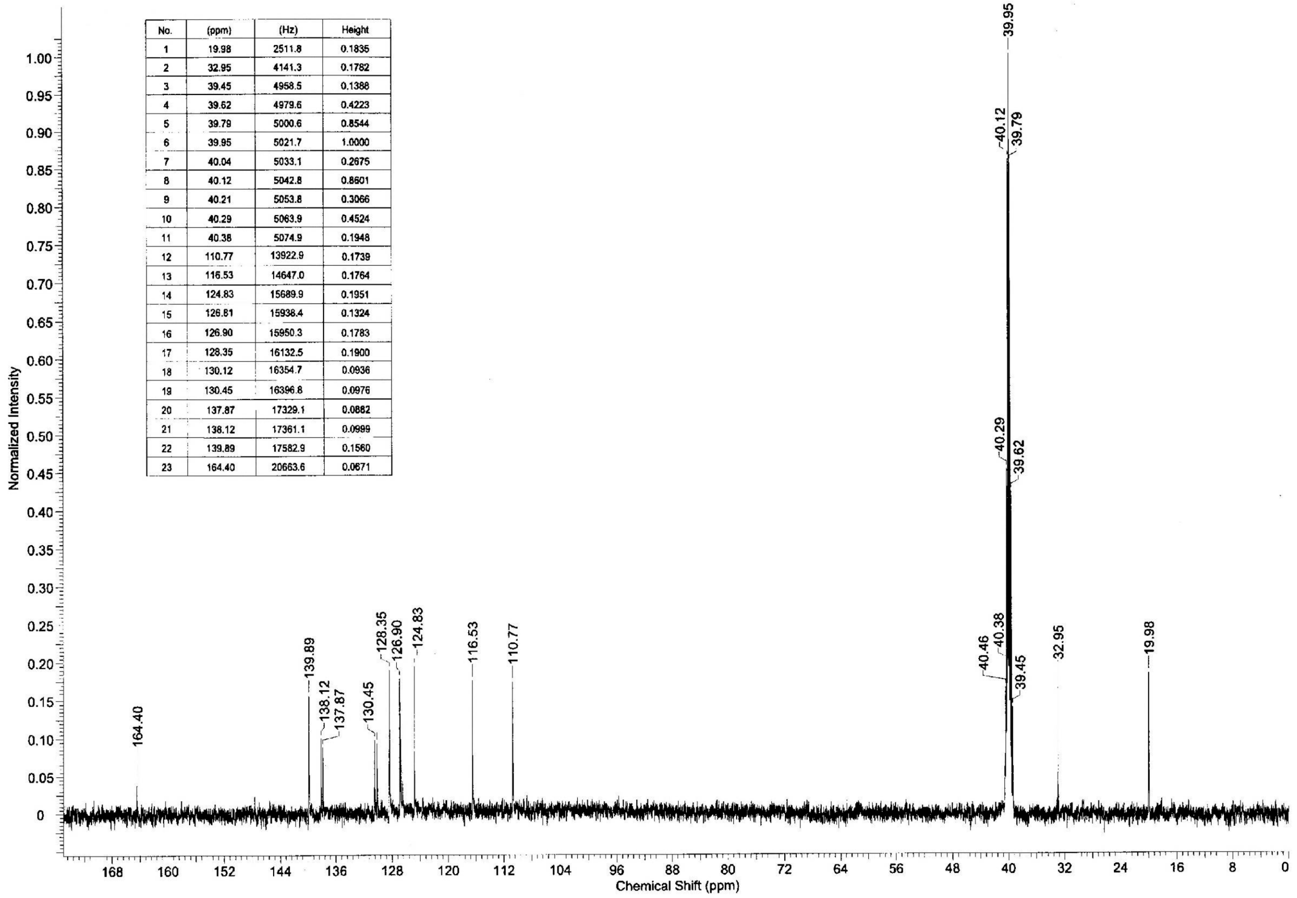

No.	(ppm)	(Hz)	Height
1	19.98	2511.8	0.1835
2	32.95	4141.3	0.1782
3	39.45	4958.5	0.1388
4	39.62	4979.6	0.4223
5	39.79	5000.6	0.8544
6	39.95	5021.7	1.0000
7	40.04	5033.1	0.2675
8	40.12	5042.8	0.8601
9	40.21	5053.8	0.3066
10	40.29	5063.9	0.4524
11	40.38	5074.9	0.1948
12	110.77	13922.9	0.1739
13	116.53	14647.0	0.1764
14	124.83	15689.9	0.1951
15	126.81	15938.4	0.1324
16	126.90	15950.3	0.1783
17	128.35	16132.5	0.1900
18	130.12	16354.7	0.0936
19	130.45	16396.8	0.0976
20	137.87	17329.1	0.0882
21	138.12	17361.1	0.0999
22	139.89	17582.9	0.1560
23	164.40	20663.6	0.0671

Spectrum 14. ^{13}C-NMR of compd **12** (125 MHz, DMSO-d_6).

SPECTRAL LINES FOR TH= 8.44 RFL= 560.1 RFP= 499.9			
INDEX	FREQ	PPM	INTENSITY
01	1675.78	8.380	52.965
02	1611.86	8.060	50.765
03	1608.33	8.043	54.788
04	1574.11	7.872	38.065
05	1570.19	7.852	40.106
06	1464.71	7.324	39.116
07	1460.76	7.305	36.583
08	730.41	3.653	145.610
09	667.32	3.337	220.732
10	502.32	2.512	39.414
11	500.45	2.503	53.137
12	498.63	2.493	40.445
13	496.93	2.485	21.817
14	489.87	2.450	108.114

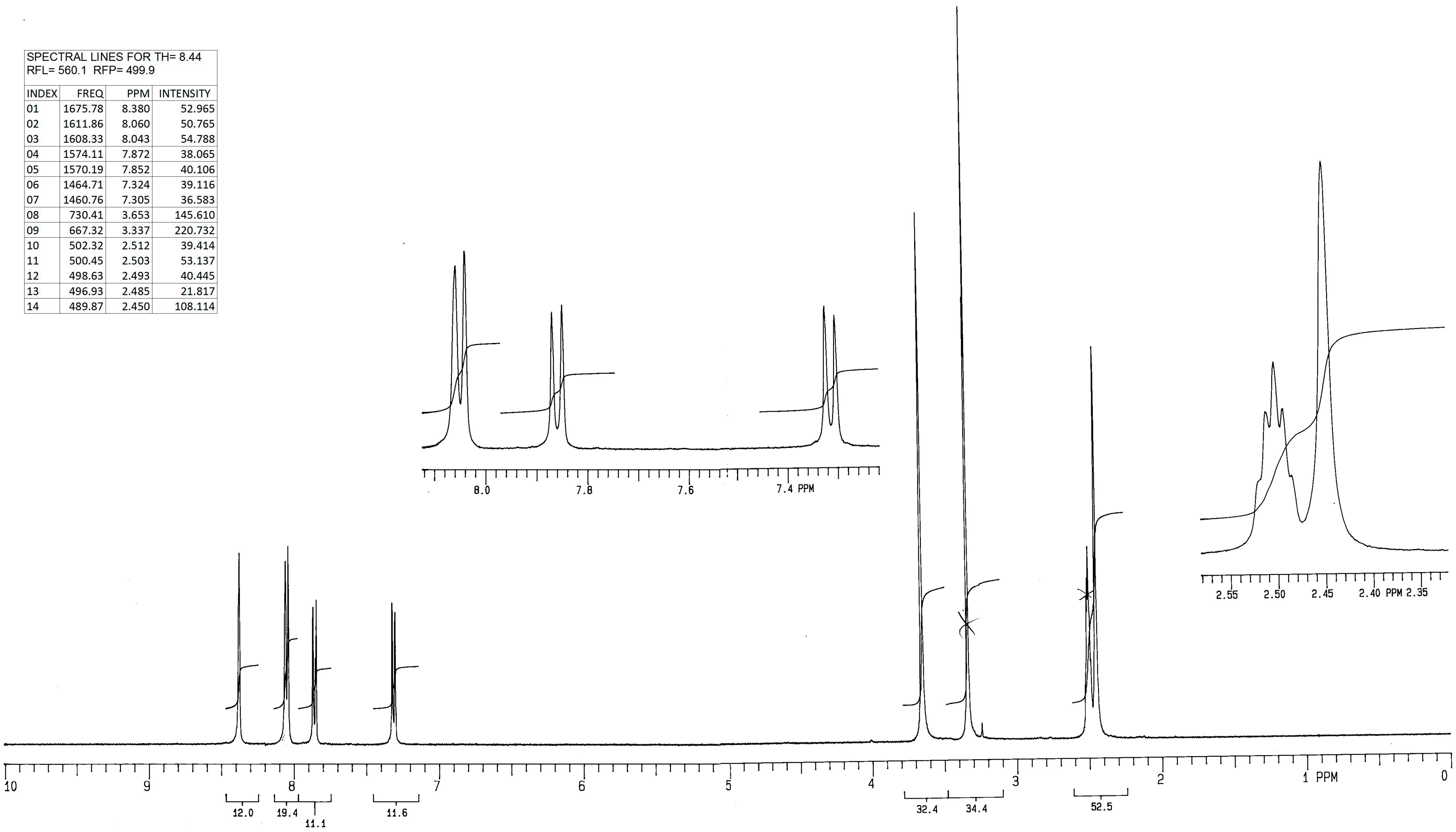

Spectrum 15. ^{1}H-NMR of compd **13** (200 MHz, DMSO-d_6).

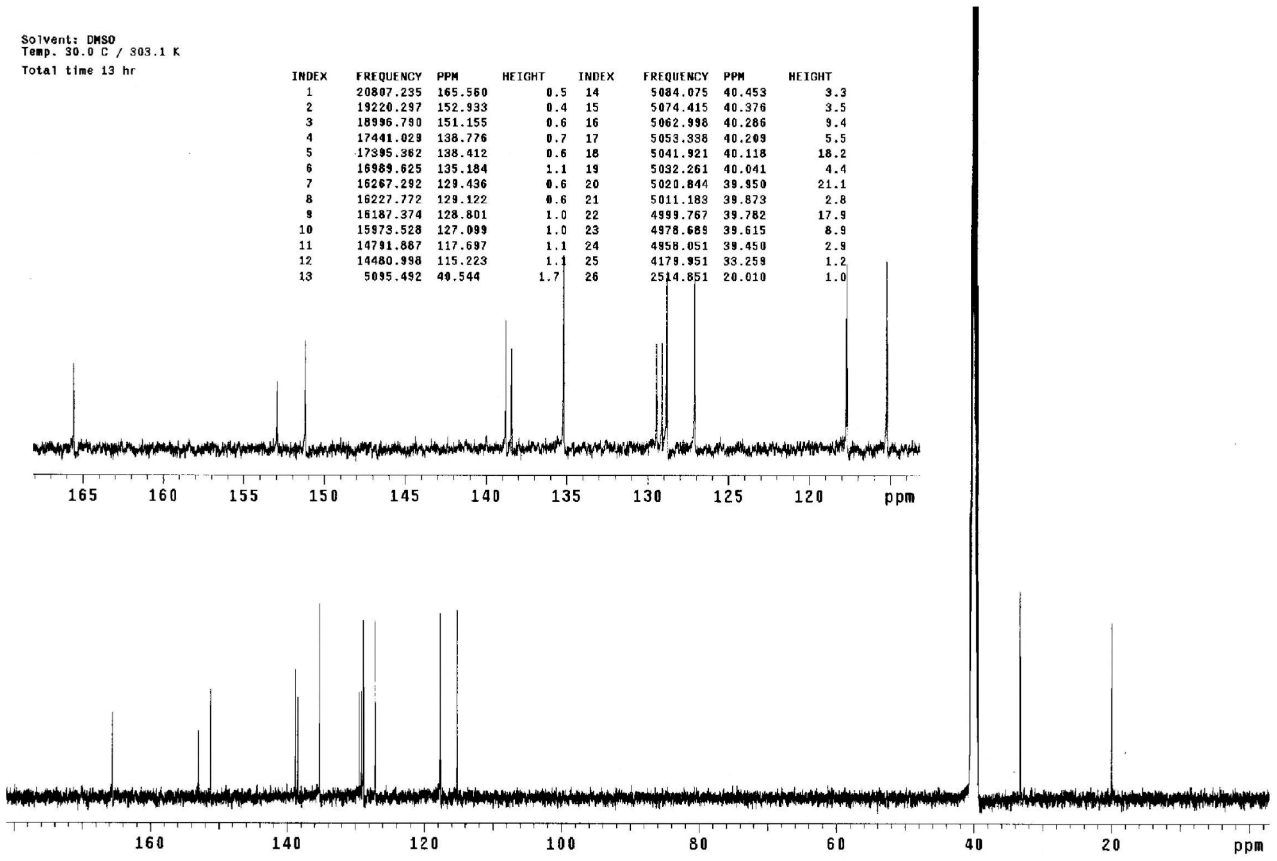

INDEX	FREQUENCY	PPM	HEIGHT	INDEX	FREQUENCY	PPM	HEIGHT
1	20807.235	165.560	0.5	14	5084.075	40.453	3.3
2	19220.297	152.933	0.4	15	5074.415	40.376	3.5
3	18996.790	151.155	0.6	16	5062.998	40.286	9.4
4	17441.029	138.776	0.7	17	5053.338	40.209	5.5
5	17395.362	138.412	0.6	18	5041.921	40.118	18.2
6	16989.625	135.184	1.1	19	5032.261	40.041	4.4
7	16267.292	129.436	0.6	20	5020.844	39.950	21.1
8	16227.772	129.122	0.6	21	5011.183	39.873	2.8
9	16187.374	128.801	1.0	22	4999.767	39.782	17.9
10	15973.528	127.099	1.0	23	4978.689	39.615	8.9
11	14791.887	117.697	1.1	24	4958.051	39.450	2.9
12	14480.998	115.223	1.1	25	4179.951	33.259	1.2
13	5095.492	40.544	1.7	26	2514.851	20.010	1.0

Spectrum 16. ^{13}C-NMR of compd **13** (125 MHz, DMSO-d_6).

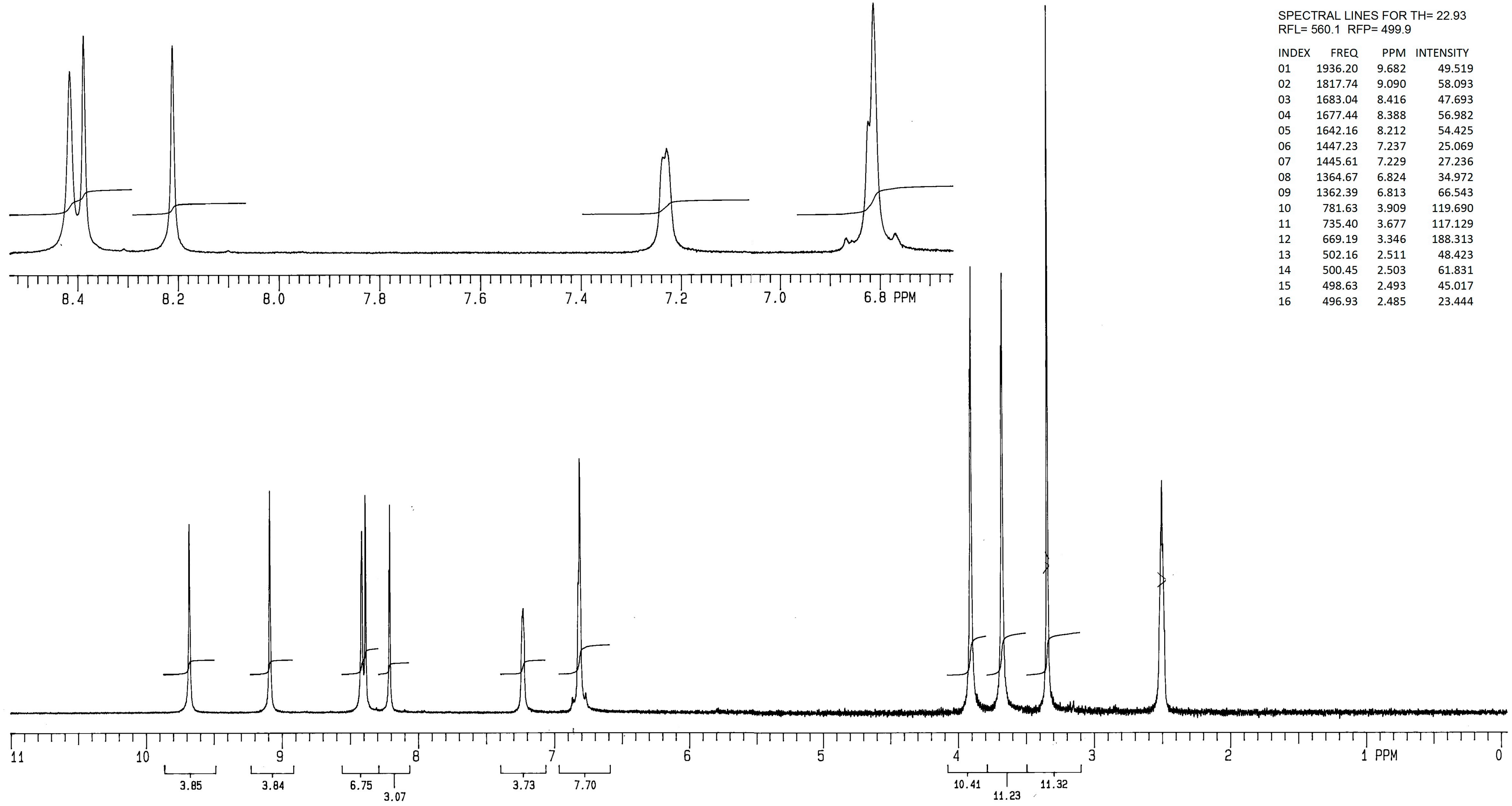

Spectrum 17. ^{1}H-NMR of compd **16** (200 MHz, DMSO-d_6).

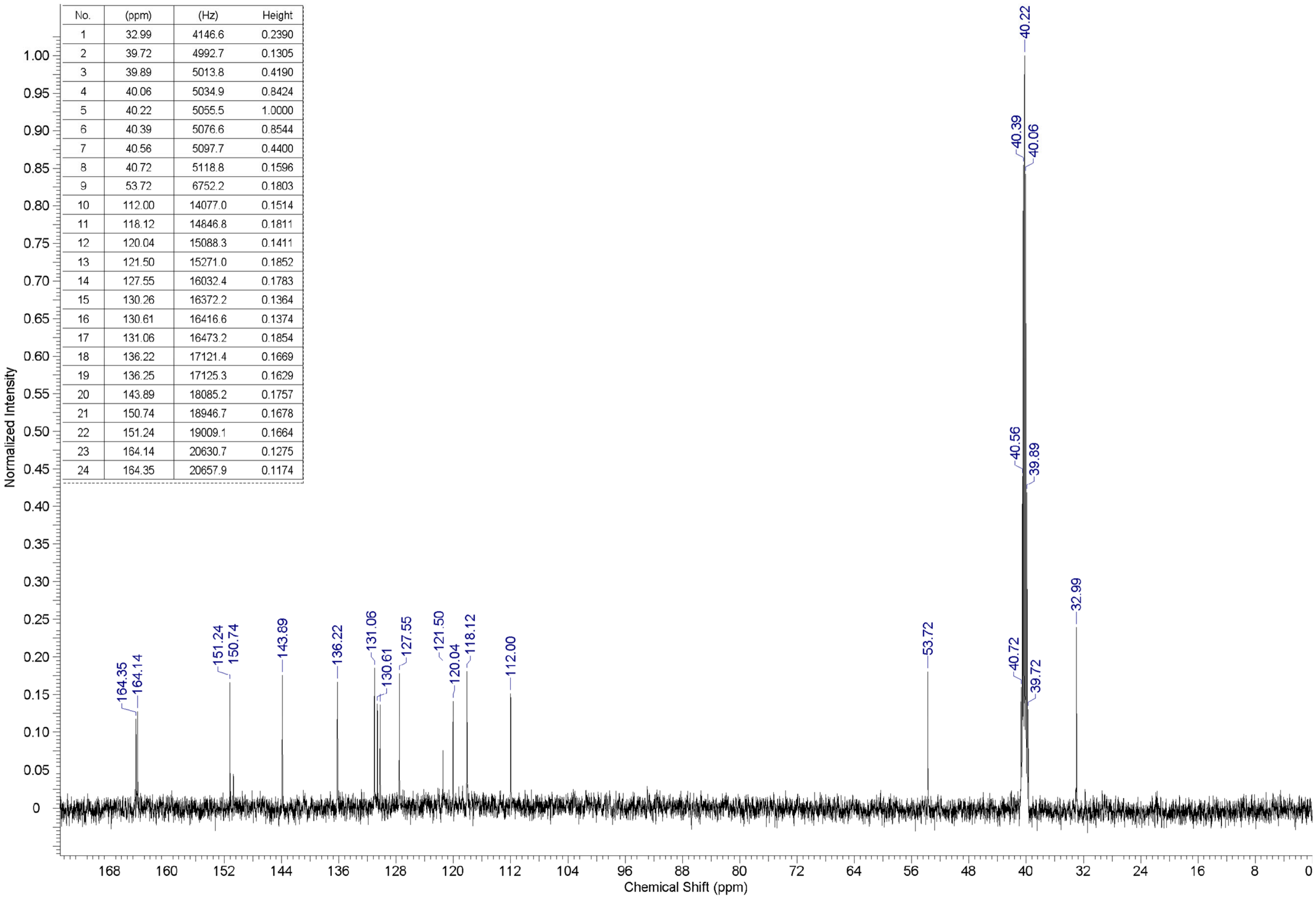

No.	(ppm)	(Hz)	Height
1	32.99	4146.6	0.2390
2	39.72	4992.7	0.1305
3	39.89	5013.8	0.4190
4	40.06	5034.9	0.8424
5	40.22	5055.5	1.0000
6	40.39	5076.6	0.8544
7	40.56	5097.7	0.4400
8	40.72	5118.8	0.1596
9	53.72	6752.2	0.1803
10	112.00	14077.0	0.1514
11	118.12	14846.8	0.1811
12	120.04	15088.3	0.1411
13	121.50	15271.0	0.1852
14	127.55	16032.4	0.1783
15	130.26	16372.2	0.1364
16	130.61	16416.6	0.1374
17	131.06	16473.2	0.1854
18	136.22	17121.4	0.1669
19	136.25	17125.3	0.1629
20	143.89	18085.2	0.1757
21	150.74	18946.7	0.1678
22	151.24	19009.1	0.1664
23	164.14	20630.7	0.1275
24	164.35	20657.9	0.1174

Spectrum 18. ^{13}C-NMR of compd **16** (125 MHz, DMSO-d_6).

SPECTRAL LINES FOR TH= 5.62
RFL= 560.1 RFP= 499.9

INDEX	FREQ	PPM	INTENSITY
01	1732.44	8.663	21.741
02	1688.65	8.444	26.558
03	1686.78	8.435	24.613
04	1575.12	7.877	16.652
05	1571.19	7.857	17.554
06	1469.43	7.348	16.994
07	1465.54	7.329	16.189
08	734.36	3.672	62.020
09	664.18	3.321	157.584
10	503.82	2.519	13.314
11	502.28	2.512	24.849
12	500.45	2.503	32.548
13	498.59	2.493	23.047
14	496.93	2.485	10.465

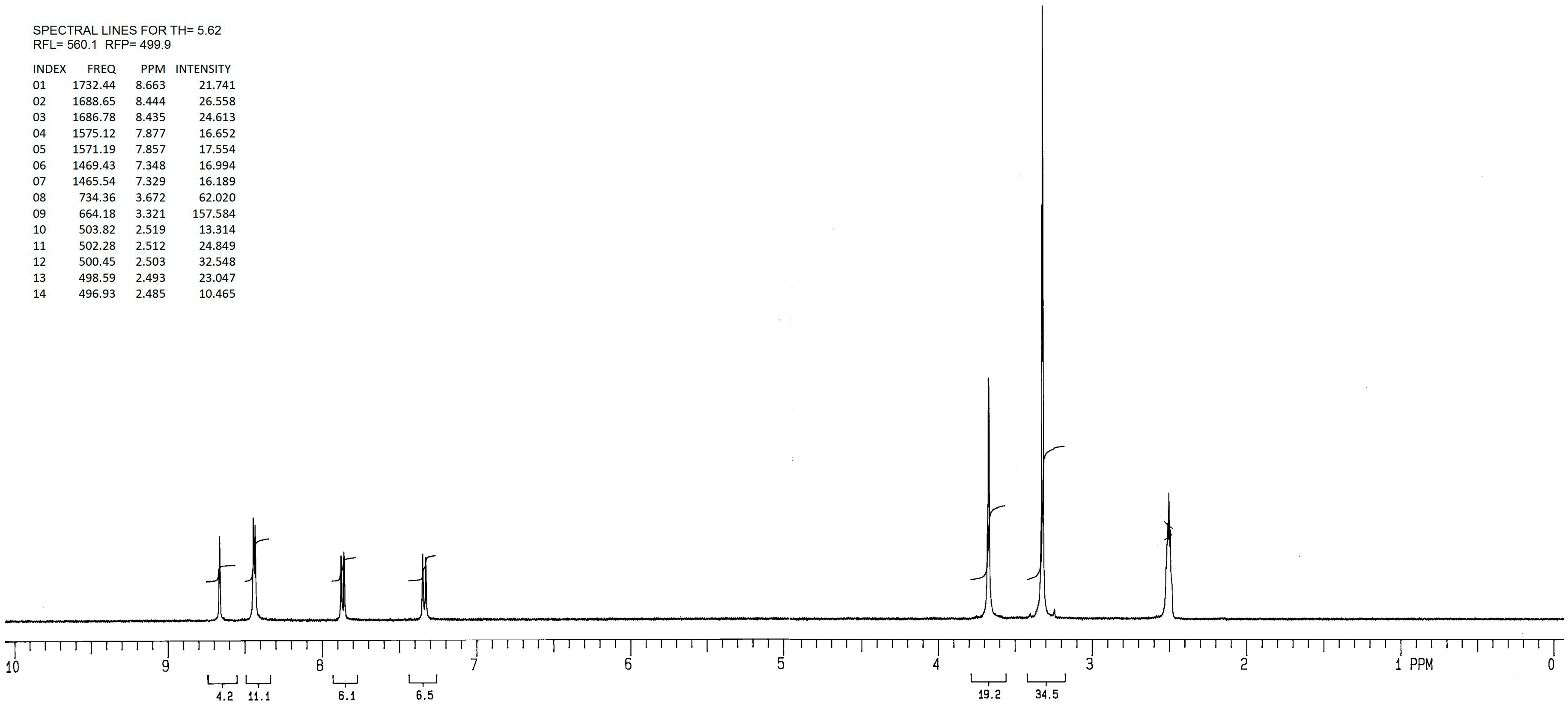

Spectrum 19. ^{1}H-NMR of compd **18** (200 MHz, DMSO-*d*6).

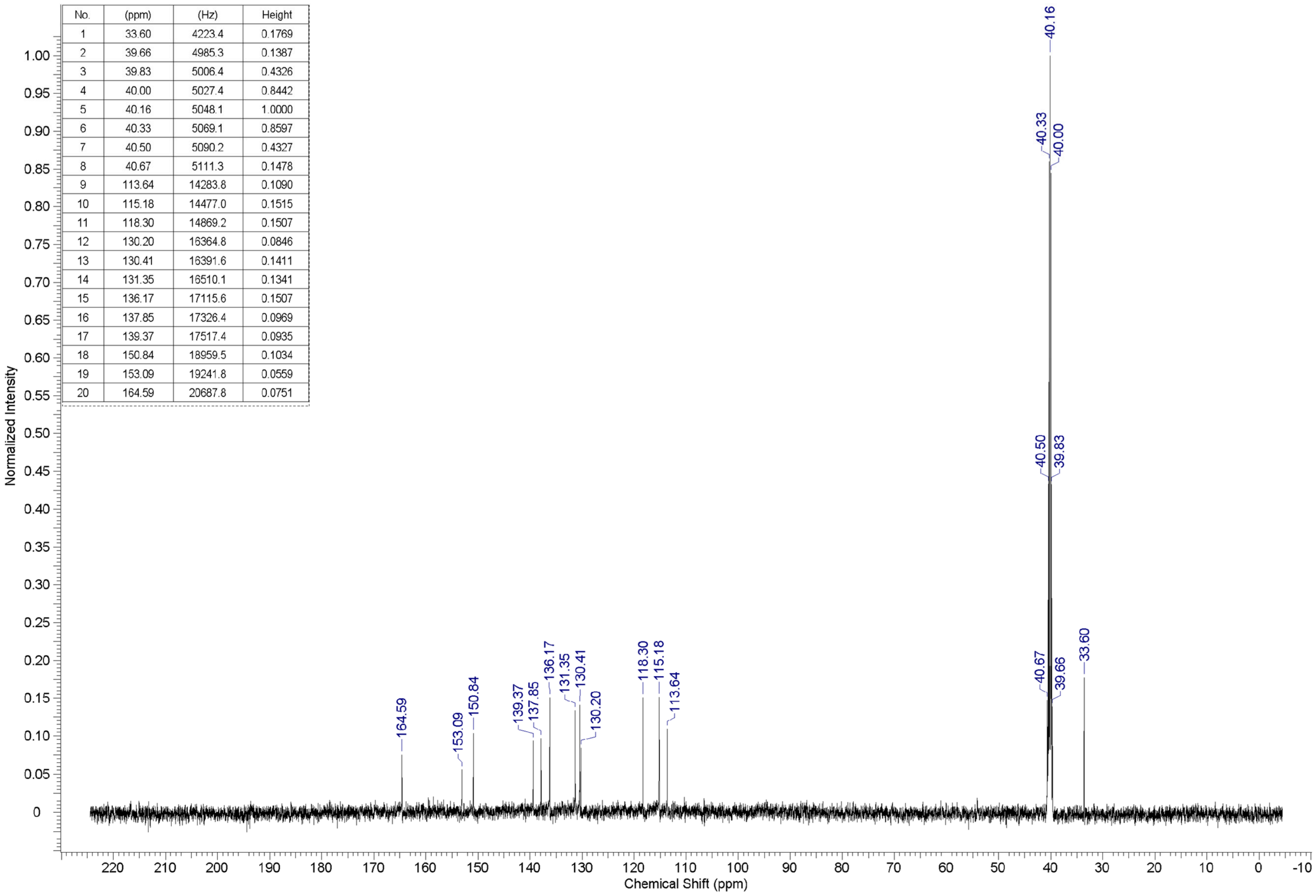

No.	(ppm)	(Hz)	Height
1	33.60	4223.4	0.1769
2	39.66	4985.3	0.1387
3	39.83	5006.4	0.4326
4	40.00	5027.4	0.8442
5	40.16	5048.1	1.0000
6	40.33	5069.1	0.8597
7	40.50	5090.2	0.4327
8	40.67	5111.3	0.1478
9	113.64	14283.8	0.1090
10	115.18	14477.0	0.1515
11	118.30	14869.2	0.1507
12	130.20	16364.8	0.0846
13	130.41	16391.6	0.1411
14	131.35	16510.1	0.1341
15	136.17	17115.6	0.1507
16	137.85	17326.4	0.0969
17	139.37	17517.4	0.0935
18	150.84	18959.5	0.1034
19	153.09	19241.8	0.0559
20	164.59	20687.8	0.0751

Spectrum 20. ^{13}C-NMR of compd **18** (125 MHz, DMSO-d_6).
